# Visual Analytic Tools and Techniques in Population Health and Health Services Research: Scoping Review

**DOI:** 10.2196/17892

**Published:** 2020-12-03

**Authors:** Jawad Ahmed Chishtie, Jean-Sebastien Marchand, Luke A Turcotte, Iwona Anna Bielska, Jessica Babineau, Monica Cepoiu-Martin, Michael Irvine, Sarah Munce, Sally Abudiab, Marko Bjelica, Saima Hossain, Muhammad Imran, Tara Jeji, Susan Jaglal

**Affiliations:** 1 Rehabilitation Sciences Institute Faculty of Medicine University of Toronto Toronto, ON Canada; 2 Advanced Analytics Canadian Institute for Health Information Toronto, ON Canada; 3 Ontario Neurotrauma Foundation Toronto, ON Canada; 4 Toronto Rehabilitation Institute University Health Network Toronto, ON Canada; 5 Universite de Sherbrooke Quebec, QC Canada; 6 School of Public Health and Health Systems University of Waterloo Waterloo, ON Canada; 7 Department of Health Research Methods, Evidence and Impact McMaster University Hamilton, ON Canada; 8 Centre for Health Economics and Policy Analysis McMaster University Hamilton, ON Canada; 9 Library & Information Services University Health Network Toronto, ON Canada; 10 Data Intelligence for Health Lab Cumming School of Medicine University of Calgary Calgary, AB Canada; 11 Department of Mathematics University of British Columbia Vancouver, BC Canada; 12 British Columbia Centre for Disease Control Vancouver, BC Canada; 13 Department of Occupational Science and Occupational Therapy University of Toronto Toronto, ON Canada; 14 Institute of Health Policy, Management and Evaluation University of Toronto Toronto, ON Canada; 15 Department of Physical Therapy Faculty of Medicine University of Toronto Toronto, ON Canada; 16 Department of Epidemiology and Public Health Health Services Academy Islamabad Pakistan

**Keywords:** visual analytics, machine learning, data visualization, data mining, population health, health services research, mobile phone

## Abstract

**Background:**

Visual analytics (VA) promotes the understanding of data with visual, interactive techniques, using analytic and visual engines. The analytic engine includes automated techniques, whereas common visual outputs include flow maps and spatiotemporal hot spots.

**Objective:**

This scoping review aims to address a gap in the literature, with the specific objective to synthesize literature on the use of VA tools, techniques, and frameworks in interrelated health care areas of population health and health services research (HSR).

**Methods:**

Using the 2018 PRISMA-ScR (Preferred Reporting Items for Systematic Reviews and Meta-Analyses Extension for Scoping Reviews) guidelines, the review focuses on peer-reviewed journal articles and full conference papers from 2005 to March 2019. Two researchers were involved at each step, and another researcher arbitrated disagreements. A comprehensive abstraction platform captured data from diverse bodies of the literature, primarily from the computer and health sciences.

**Results:**

After screening 11,310 articles, findings from 55 articles were synthesized under the major headings of visual and analytic engines, visual presentation characteristics, tools used and their capabilities, application to health care areas, data types and sources, VA frameworks, frameworks used for VA applications, availability and innovation, and co-design initiatives. We found extensive application of VA methods used in areas of epidemiology, surveillance and modeling, health services access, use, and cost analyses. All articles included a distinct analytic and visualization engine, with varying levels of detail provided. Most tools were prototypes, with 5 in use at the time of publication. Seven articles presented methodological frameworks. Toward consistent reporting, we present a checklist, with an expanded definition for VA applications in health care, to assist researchers in sharing research for greater replicability. We summarized the results in a Tableau dashboard.

**Conclusions:**

With the increasing availability and generation of big health care data, VA is a fast-growing method applied to complex health care data. What makes VA innovative is its capability to process multiple, varied data sources to demonstrate trends and patterns for exploratory analysis, leading to knowledge generation and decision support. This is the first review to bridge a critical gap in the literature on VA methods applied to the areas of population health and HSR, which further indicates possible avenues for the adoption of these methods in the future. This review is especially important in the wake of COVID-19 surveillance and response initiatives, where many VA products have taken center stage.

**International Registered Report Identifier (IRRID):**

RR2-10.2196/14019

## Introduction

### Background

Visual analytics (VA) is a term that was formally introduced in the literature 15 years ago [1,2]. It describes a semiautomated approach to electronic data processing, guided by users who are able to interact with data through an interface [3,4]. In essence, VA transforms large amounts of quantitative or qualitative information into graphical formats that can be modified based on the operator’s needs [4,5]. The resulting views can be used by users with diverse backgrounds to better understand data, communicate results, and disseminate information across a broad spectrum of disciplines [6,7].

The implementation and use of VA have bloomed in many sectors of health care systems during the past decade [8]. Population health research involves the study of data related to health outcomes and determinants among and between populations [9,10], whereas health services research (HSR) explores the functioning of the health care system and its workforce in relation to access, quality, costs, and patient outcomes [11,12]. Both fields involve the analysis of big data, including information collected through clinical databases, administrative data sets, or electronic health records (EHRs) [13-15]. VA offers the opportunity for health data users, such as clinicians, researchers, decision makers, and consumers, to visually explore and interpret complex data sets to guide decision making and knowledge discovery [3,16].

### Rationale

Although researchers have pointed out the lack of literature on the extent of the use of VA applications in various sectors [3], we identified 4 recent systematic reviews that covered varied areas of VA applications in health care. The 2018 paper by Islam et al [17] was one of the most comprehensive reviews about data mining applications in health care. However, the review is limited to mining approaches for health care data and does not primarily cover VA. The recently published review by Chung et al [8] relates to VA approaches in mental health care systems and policy. One of the most cited systematic reviews is that by West et al [18] on the use of visualization for EHRs aimed at knowledge discovery. Although these reviews cover some aspects of the wide field of visualization and analytics in health care, none have focused on areas of population health and HSR.

One of the seemingly close literature syntheses is the review by Wu et al [19] on visualization and VA technologies in medical informatics for characterizing evaluation methods. However, there are significant distinctions between that paper and our review. First, their review [19] relates to the subject area of health informatics, which is almost exclusively concerned with patient data in the context of care provision. The classic definition of the subject area is “the applications of information technology to healthcare delivery” [20]. Second, Wu et al [19] cover evaluation methods for VA applications and not VA applications themselves. Our scoping review focuses on methods related to VA applications in population health and HSR and does not focus on evaluation methods.

Through this review, we attempt to bridge a critical gap in the literature on the use of VA tools, techniques, and frameworks in the interrelated and overlapping areas of population health and HSR. To the best of our knowledge, none of the recent systematic literature syntheses focused on these areas of health care or covered the VA tools and techniques that we present in this scoping review.

In response to this broader conceptualization, this scoping review identified and synthesized findings from English language peer-reviewed sources that used VA approaches and methods in population health and HSR. Such a synthesis of the literature will be helpful for researchers, practitioners, and decision support analysts to (1) explore recent trends in the use of innovative VA methods in the important health care domains of population health and HSR, (2) learn from methodological frameworks, and (3) uptake these techniques to meet the growing needs for data-driven insights. Furthermore, this review presents the settings for which VA applications are developed and applied as well as the intended target audience. This information is important in the context of the use of VA techniques in participatory co-design initiatives.

### Objectives

The objectives of this review are (1) to identify the scope and nature of the use of VA methods in population health and HSR and (2) to summarize methodological tools, techniques, and frameworks from peer-reviewed literature in both health care areas.

## Methods

### Protocol and Overall Scoping Review Methodology

The study protocol was previously published, detailing the search strategy and methods [21]. We primarily followed the Joanna Briggs Institute guidelines on scoping reviews [22] and the framework by Arksey and O’Malley for conducting scoping reviews [23], with improvements suggested by Levac et al [24] and Peters et al [25] for conceptualizing the population, concepts, and context of the study, especially given the context of a methods-based review.

We further used the Preferred Reporting Items for Systematic Reviews and Meta-Analyses Extension for Scoping Reviews (PRISMA-ScR) checklist from the work by Tricco et al [26] to operationalize the different steps, while providing milestones and guideposts for adaptation during the review. The checklist is shown in Multimedia Appendix 1. We followed the journal guidelines for the preparation of the manuscript. The major methodological steps for the systematic scoping review comprised determining the research question; identifying relevant studies; title, abstract, and full-text screening; data abstraction; and the collation, summarization, and reporting of the results.

### Eligibility Criteria

The inclusion and exclusion criteria are presented in Textboxes 1 and 2, respectively. Papers included during the screening stage needed to have a central VA component with a focus on population health or HSR. Studies conducted in clinical settings or focusing on a single condition, without a population or health service component, were not included in the review. The operational definitions for all concepts are presented in detail later in this section.

**Inclusion criteria for selection of articles.**

Inclusion criteria

- Peer-reviewed or conference papers
- January 1, 2005, to March 31, 2019
- Population health or health services research (HSR) related
- Articles with population-level or HSR metrics: incidence, prevalence, events over time and space, spatiotemporal, access, utilization, disease or condition distribution, and social or multiple determinants of health
- Articles with an analytic engine and a visualization engine
- Articles with exploratory data analytic techniques
- Articles on electronic medical records and electronic health records
- Articles with dashboards with an explicit analytic engine to feed data
- Articles with automated analysis, data mining techniques, interactive tools, and iterative analysis

**Exclusion criteria for the articles.**

Exclusion criteria

- Articles not in the English language
- Editorials, projects, reviews, book chapters, short papers, or reports
- Articles on medical imaging
- Studies conducted in clinical settings without a population-level or health services research component
- Articles for individual condition from a single hospital or unit, such as intensive care, surgery, anesthesia, without a population-level or health services research (HSR) component
- Articles on device or sensor data without a population-level or HSR component
- Studies lacking an analytic method or engine
- Cartographic or geographic information systems (GIS) method

One of the primary aims of both population health and HSR is to better understand disease distribution and barriers to equitable care. We included these components and related metrics for population health, such as incidence, prevalence, and events over time and space, to guide us in delineating research that focused on clinical or individual conditions or cases. For example, if a diabetes dashboard presented clinical care for an individual in a hospital setting, such as blood sugar or glycated hemoglobin levels, it was excluded. However, if a diabetes dashboard presented a *population* with glycated hemoglobin levels in a hospital catchment area, it was included as it had a population-level component. Studies without an analytic engine were excluded. Finally, articles not in the English language and non–peer-reviewed work, such as editorials, projects, short papers, conference abstracts, and reports, were excluded.

**Figure 1:**
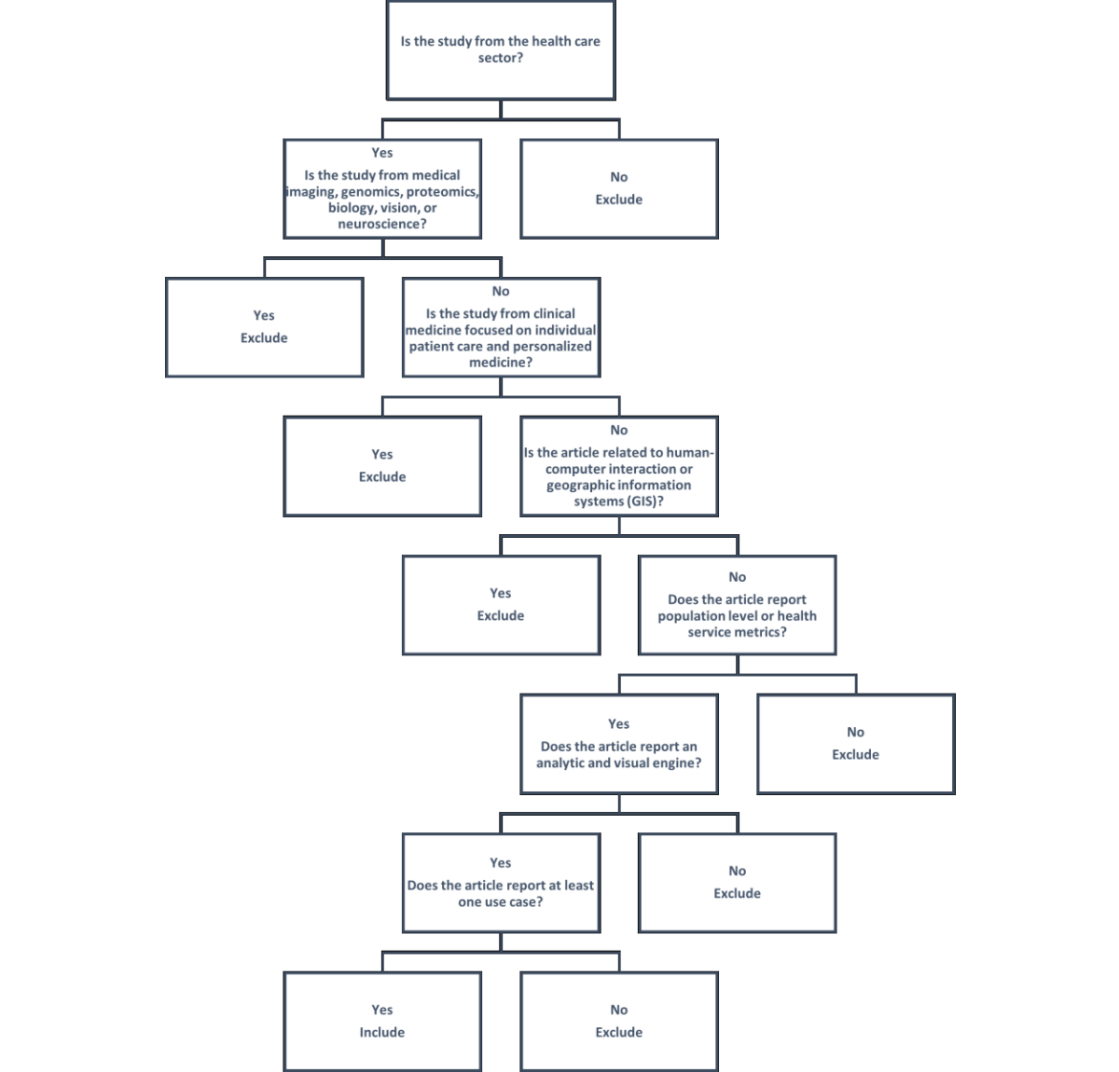
Decision tree for assessing article eligibility.

The eligibility criteria were revised twice during the screening process. In total, 4 items were added later to the exclusion criteria: studies conducted in clinical settings without a population-level component, articles on device or sensor data, articles related to cartographic methods, and articles related to geographic information systems (GIS) techniques. However, VA articles with a GIS component covering spatiotemporal data, sometimes termed geo-VA, were included in the review. Figure 1 shows a simplified decision tree for the selection of articles.

### Scoping Review Timeline

We were able to trace the first formal use of the term VA to the seminal work by Thomas and Cook in 2005 [2,3]. As the use of the term was in the area of national defense, we expected a lag time in the adoption of the methodology and the use of the term in health care. On the basis of these reasons, our multidisciplinary team decided to include articles from January 1, 2005, to March 31, 2019.

### Information Sources

The full electronic search strategy is provided in Multimedia Appendix 2. It was developed through an iterative process by the research team, which included an information specialist (JB). A preliminary search was conducted in MEDLINE, following which the first 100 resulting article abstracts were reviewed to refine the search strategy. The search strategy was then peer reviewed using the Peer Review of Electronic Search Strategies (PRESS) guidelines by a second information specialist. A total of 6 databases were searched in April 2019 using both keywords and subject-specific vocabulary (eg, Medical Subject Headings [MeSH], Emtree; Table 1). We have detailed the search strategy, the keywords used, and the operationalization of the concepts in depth in the published protocol [21].

**Table 1:** Databases and search results.

Database	Platform	Search results (n=14,099), n
MEDLINE	OvidSP	4633
EMBASE	OvidSP	1880
Web of Science core collection	Web of Science	5396
Compendex	Engineering Village	1267
IEEE Xplore	IEEE	151
Inspec	Engineering Village	772

The review management software Covidence was used to manage the search results, including the importing of references, screening of citations, and conflict resolution [27]. Duplicates were removed in 3 phases. First, citations were checked in EndNote (Clarivate Analytic) for duplicates, followed by duplicate identification by Covidence (Veritas Health Innovation) systematic review software. Finally, duplicates were removed manually during the full-text review.

To complement the database searches, we conducted an internet search using Google and Google Scholar search engines, and we manually searched 10 journals deemed relevant to the research question. These were *Applied Clinical Informatics*, *Visual Analytics in Healthcare Proceedings,* *IEEE Transactions on Information Technology in Biomedicine*, *Journal of Medical Internet Research*, *Journal of Medical Systems*, *Journal of the American Medical Informatics Association*, *Health Affairs*, *Journal of Biomedical Informatics*, *Healthcare Informatics Research*, and *PLOS One*. We further reviewed the conference proceedings from Visual Analytics in Health Care, which is held one year apart in collaboration with the American Medical Information Association and IEEE VIS conferences. In addition, we reviewed the references from another 13 systematic and narrative topic-related reviews identified during the screening of the articles [7,16-19,28-35].

### Selection of Sources of Evidence

**Figure 2:**
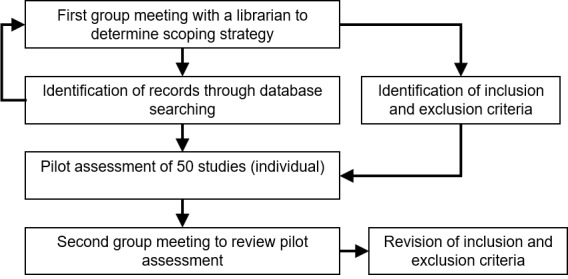
Pilot assessment and revision of criteria for selection of sources of evidence.

The process for the selection of sources of evidence was divided into 2 phases. First, to enhance the consistency among reviewers in the team, we met to discuss the inclusion and exclusion criteria. We randomly selected 50 articles that each reviewer screened for title and abstract. After this initial pilot assessment, we discussed the process, criteria, conflicts, ambiguities, and difficulties encountered. This pilot phase led to a slight readjustment of the inclusion and exclusion criteria. This iterative methodology, with the selection of sources of evidence is illustrated in Figure 2. In the second phase, 2 reviewers were required for the title and abstract screening process as well as for the full-text screening process. In both cases, conflicts were resolved by another reviewer.

### Data Charting

The data charting form was developed specifically for this review and piloted with 3 randomly selected articles to refine the categories of abstraction. Each article was then assigned to 2 independent researchers. A third researcher offered arbitration, correction, and validation of the abstraction where required. For the specific abstraction fields, we followed the definitions detailed in the *Operational Concepts and Definitions* section.

### Data Items and Synthesis of Results

Data abstraction was based on 5 major categories: (1) study characteristics (eg, country, problem, settings, target audience); (2) frameworks, tools, and techniques used (eg, tool name, framework followed); (3) analytic and visualization methods and engines used (eg, analytic engine, data type, data used); (4) domains of health care and type of measures used (eg, population health, health services); and (5) study innovation, impact, availability of the tool, and whether it was co-designed with the target audience. The results were compiled into tables under these major categories, following the major schema developed during the operationalization of the concepts and abstraction of the articles.

### Operational Concepts and Definitions

Our initial literature search revealed varying definitions and inconsistent use of terms for the 3 major concepts: population health, HSR, and VA. To translate these terms into operational definitions, we undertook a 3-pronged strategy of studying seminal literature, recent systematic reviews, and subject trees in MEDLINE. Although the search terms and their sources are detailed in the study protocol [21], we detail the operationalized concepts below.

#### Population Health and HSR

Defined as the “science and art of preventing disease” [36], population health is nested under the larger concept of public health. Although experts have attempted to develop a common language related to public and population health [37], population health as a MeSH term was only recently added to MEDLINE in 2018 [38]. Kindig and Stoddart [9,10] define population health as “the health outcomes of a group of individuals, including the distribution of such outcomes within the group” that includes “health outcomes, patterns of health determinants, and policies and interventions that link these two.” We found the expanded definition of population health by Kindig and Stoddart [9,10] apt for our review to encompass the vast nature of this area. To adapt search terms, we studied the national public health language created by the National Institute for Health and Care Excellence in the United Kingdom [39], detailed database trees, and 5 recent reviews [40-44].

HSR is defined by the Canadian Institutes of Health Research as research with the “goal of improving the efficiency and effectiveness of health professionals and the health care system” [11]. Population health and HSR are intertwined concepts, with overlapping research and communities of practice, first, in the purview of studying problems through a population lens and, second, through a health systems and service lens. The *population health approach* brings together the two in their application toward health sector reform, allowing researchers to formulate proposals for the organization and delivery of health care [41,45].

Guided by 4 recent reviews [45-48] and the filters for HSR developed by the National Library of Medicine [49], we translated the concept to the search strategy. Particular to HSR, we included studies on access, utilization, and cost of health services in the review.

#### VA: Analytic and Interactive Visual Engines

The seminal work by Thomas and Cook [2] defines VA as “the science of analytical reasoning facilitated by interactive visual interfaces.” Later, Keim et al [3] extended this concept to “automated analysis techniques with interactive visualizations for an effective understanding, reasoning and decision making on the basis of very large and complex data sets.” Although these definitions offered a high-level conceptualization of the expansive field of VA, we needed a simplified, more encompassing conceptual definition that could help contextualize VA methods and applications in health care. Hence, we opted to use the expanded definition of VA applications in health care by Ola and Sedig [50], comprising analytic and interactive visualization engines. Typically, the analytics engine involves data storage, transformation, and analysis, whereas the visualization engine provides functionality toward data manipulation and display [50].

The analytics engine can employ advanced statistical and machine learning (ML) techniques for various functions. For example, an extract, transform, and load engine using ML algorithms can bring together a database that the visual engine uses to produce visualizations [50]. For the purposes of the review and its focus on population health and HSR, we avoided the term artificial intelligence.

ML is a subset of artificial intelligence methods that includes fitting models to data and learning by training models with data [51]. We focused on tasks such as clustering, classification, and algorithms used to present the major techniques used toward the analytic engine.

Interactivity is one of the recent hallmarks of VA applications, owing to the manipulation of visual interfaces afforded by computing power [50]. We borrow from works by Ola and Sedig [50] and Pike et al [52] to define *interactivity* as the ability to reflect changes in the visual representation of data based on one or more indicators available on the analytic interface to the user. Pike et al [52] categorize interaction elements into 2 main types: *lower level* aimed at change of the visual representation to study patterns, relationships, and the like and *higher level* that offers an understanding of the intent of interaction itself toward knowledge discovery. To select the appropriate literature as part of this scoping review, we focused on lower-level interaction that includes tasks such as filtering, determining ranges, finding anomalies, clustering, and the like by providing menus, dropdowns, and other options on the visualization interface.

Furthermore, to operationalize the search terms related to VA, we studied 4 recent reviews [7,17-19] in addition to 9 seminal papers [6,16,53-59].

#### Analytic Types and Capability, Settings, and Target Audience

To operationalize the types of analytics that the application targeted within the use case, we adapted the work by Islam et al [17] on data mining techniques in health care. Analytics is defined as “knowledge discovery by analyzing, interpreting and communicating data” [17].

Related to the analytic capability, applications were categorized as being primarily meant for descriptive, predictive, or prescriptive analytics for visual exploration of complex data sets or a combination of these. Descriptive analytics is defined as “exploration and discovery of information in the dataset,” predictive analytics is defined as “prediction of upcoming events based on historical data,” and prescriptive analytics is defined as “utilization of scenarios to provide decision support” [17]. Although the visual exploration of complex data sets can be seen as an extension of descriptive analytic capability, we kept it as a separate category.

We gleaned information from different parts of the included articles to obtain the study setting and audience based on the potential application for the method, tool, or its user, as mentioned by the authors.

#### Tools, Applications, and Frameworks

Tools were defined as software used to develop an application to address a certain problem, whereas applications were one or more software program using code or front-end programming employed for data analysis and visualization.

Frameworks in research form the foundation, backbone, or the *blueprint* on which knowledge is constructed [60]. Hence, we opted to study the frameworks that formed the basis for the applications to better situate the literature on VA and to help define the lens, perspective, and conceptual background for the methods. We defined a framework as an extension of a lens or perspective of inquiry that is structured to allow methodological uniformity, adaptation, reporting, understanding, and replicability. Given that our review is methods based, we did not differentiate between a theoretical or conceptual framework [60].

#### Use Case and Data Source

A use case is defined as the application of the method to an actual data set, source, or simulation data related to population health or HSR. We studied whether the use case included a single data source or multiple data sources. The goal of the application was ascertained by studying whether the application, tool, or method was meant for decision support, knowledge discovery, or both.

#### Domains of Health Care

Finally, we adapted the domains of health care from Islam et al [17] to represent population health, HSR, or both. We further divided population health–related articles into clinical, demographic, epidemiologic, spatiotemporal, or a combination of these categories. The clinical category would include a condition, the demographic category would include any population-related characteristic such as age, the epidemiologic category would include disease distribution and dynamics, and the spatiotemporal category would include events over time and space. An overlap between the categories within the articles was expected.

#### Co-Design and Knowledge Translation

Knowledge translation is a wide term used in different contexts, focusing on the translation of research evidence to policies and practice [61]. Although our initial conceptualization for the review was related to knowledge co-creation for decision making, we realized that for the purpose of this review, a better approach would be to consider *co-design* methods especially in the development stages of an application. We used the definition of co-design in health care by Ward et al [62] that encompasses the partnership of health workers, patients, and designers who aspire to change, depending on shared knowledge to achieve *better outcomes or improved efficiency*.

Co-designed applications would have better viability and uptake toward both knowledge transfer and decision support. We studied whether the authors involved stakeholders or target audiences during the development of an application. We did not study co-design methods and approaches, as this was not the objective of the review.

## Results

### Selection of Articles

We identified 14,099 articles through the combined database searches. Using EndNote, 2078 duplicates were electronically removed in 6 iterations run on 2 different versions, X7 and X9. On importing 12,021 records into Covidence, another 711 duplicates were removed. We screened the titles and abstracts for 11,310 records, of which 10,819 (95.65%) were excluded. We were able to identify 57 more references from 4 systematic reviews identified during the screening process [8,17-19] and hand searching. The results are summarized in a Tableau dashboard [63].

**Figure 3:**
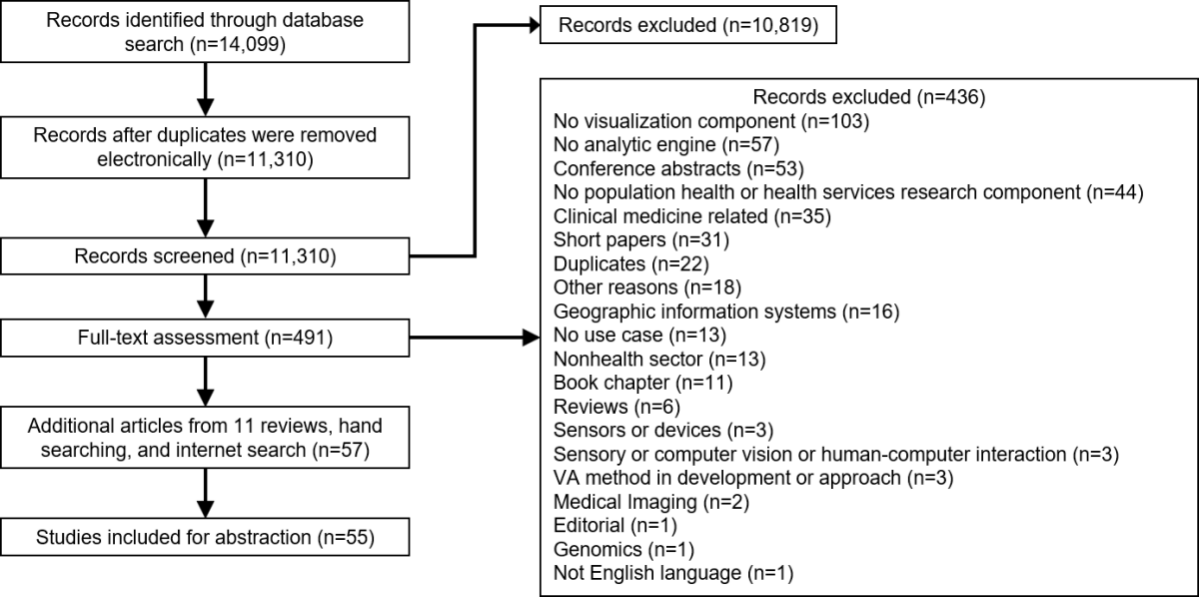
PRISMA (Preferred Reporting Items for Systematic Reviews and Meta-Analyses) chart for article selection. VA: visual analytics.

Of the 491 records included for full-text assessment, 436 (88.8%) were excluded. Reasons for exclusion were lacking a visualization component (n=103), lacking an analytic engine (n=57), conference abstracts and editorials (n=53), not population health or HSR (n=44), clinical medicine related (n=35), short papers (n=31), duplicates (n=22), cartographic and GIS methods (n=16), no use case (n=13), non–health sector (n=13), book chapter (n=11), reviews (n=6), sensors or devices (n=3), sensory or computer vision or human-computer interaction (n=3), VA method in development or approach (n=3), medical imaging (n=2), genomics (n=1), not English language (n=1), and other reasons (n=18). Overall, 55 articles were included for abstraction. The PRISMA-ScR flow diagram is shown in Figure 3.

### Study Characteristics, Settings, and Target Audience

The country of the study was ascertained from the actual use case of the VA application. The 55 studies included in the analysis were from 19 countries, including the United States (24/55, 44%), Canada (5/55, 9%), and Germany (3/55, 5%). Details are provided in Multimedia Appendix 3 [64-118].

In terms of the settings where the research took place, studies were most often conducted as part of, or with the involvement of, a government unit, including a ministry or health department (38/55, 69%), followed by academic settings (35/55, 64%), mixed government and academic settings (18/55, 33%), and the industry (3/55, 5%). The intended target audience was the population health and HSR community (53/55, 96%), academic researchers and data scientists (47/55, 85%), clinicians (21/55, 38%), both clinicians and population health and HSR practitioners (21/55, 38%), policy and decision makers (7/55, 13%), consumers and the general public (5/55, 9%), and the industry (3/55, 5%). Table 2 details the study setting, while Table 3 details the target audience in the included papers.

**Table 2:** Setting of the use cases.

Setting	Values, n (%)	Study (reference)
Academic	38 (69)	Abusharekh et al, 2015 [67]; Afzal et al, 2011 [85]; Ali et al, 2016 [68]; Alonso et al, 2012 [92]; Antoniou et al, 2010 [93]; Benis et al, 2017 [89]; Bryan et al, 2015 [64]; Byrd et al, 2016 [94]; Chorianopoulos et al, 2016 [96]; Garcia-Marti et al, 2017 [97]; Gotz et al, 2014 [76]; Guo et al, 2007 [69]; Hardisty et al, 2010 [100]; Hund et al, 2016 [90]; Ji et al, 2012 [102]; Ji et al, 2013 [81]; Jiang et al, 2016 [103]; Kaieski et al, 2016 [104]; Katsis et al, 2017 [105]; Kostkova et al, 2014 [75]; Lavrac et al, 2007 [70]; Lu et al, 2017 [71]; Luo et al, 2016 [78]; Maciejewski et al, 2010 [107]; Maciejewski et al, 2011 [79]; Marek et al, 2015 [108]; Ozkaynak et al, 2015 [111]; Park et al, 2018 [112]; Perer et al, 2015 [113]; Proulx et al, 2006 [114]; Shaban-Nejad et al, 2017 [84]; Tate et al, 2014 [87]; Widanagamaachchi et al, 2017 [72]; Xing et al, 2010 [91]; Xu et al, 2013 [73]; Yan et al, 2013 [118]; Yu et al, 2017 [82]; Yu et al, 2018 [74]
Government, ministry, or health department	35 (64)	Abusharekh et al, 2015 [67]; Afzal et al, 2011 [85]; Alonso et al, 2012 [92]; Antunes de Mendonca et al, 2015 [86]; Baytas et al, 2016 [80]; Benis et al, 2017 [89]; Bryan et al, 2015 [64]; Castronovo et al, 2009 [77]; Chen et al, 2016 [95]; Dagliati et al, 2018 [66]; Deodhar et al, 2015 [65]; Gligorijevi et al, 2017 [98]; Haque et al, 2014 [99]; Hardisty et al, 2010 [100]; Huang et al, 2015 [101]; Jiang et al, 2016 [103]; Jinpon et al, 2017 [83]; Kaieski et al, 2016 [104]; Kruzikas et al, 2014 [106]; Lavrac et al, 2007 [70]; Lu et al, 2017 [71]; Maciejewski et al, 2011 [79]; Mitrpanont et al, 2017 [109]; Mittelstadt et al, 2014 [110]; Ozkaynak et al, 2015 [111]; Proulx et al, 2006 [114]; Shaban-Nejad et al, 2017 [84]; Soulakis et al, 2015 [115]; Tilahun et al, 2014 [88]; Toddenroth et al, 2014 [116]; Torres et al, 2012 [117]; Xu et al, 2013 [73]; Yan et al, 2013 [118]; Yu et al, 2017 [82]; Yu et al, 2018 [74]
Academic and government or ministry or health department	18 (33)	Abusharekh et al, 2015 [67]; Afzal et al, 2011 [85]; Alonso et al, 2012 [92]; Benis et al, 2017 [89]; Bryan et al, 2015 [64]; Hardisty et al, 2010 [100]; Jiang et al, 2016 [103]; Kaieski et al, 2016 [104]; Lavrac et al, 2007 [70]; Lu et al, 2017 [71]; Maciejewski et al, 2011 [79]; Ozkaynak et al, 2015 [111]; Proulx et al, 2006 [114]; Shaban-Nejad et al, 2017 [84]; Xu et al, 2013 [73]; Yan et al, 2013 [118]; Yu et al, 2017 [82]; Yu et al, 2018 [74]
Industry	3 (5)	Gotz et al, 2014 [76]; Perer et al, 2015 [113]; Yu et al, 2018 [74]

**Table 3:** Target audience of the use cases.

Target audience	Values, n (%)	Study (reference)
Population or public health and health services research practitioners	53 (96)	Abusharekh et al, 2015 [67]; Afzal et al, 2011 [85]; Ali et al, 2016 [68]; Alonso et al, 2012 [92]; Antoniou et al, 2010 [93]; Baytas et al, 2016 [80]; Benis et al, 2017 [89]; Bryan et al, 2015 [64]; Byrd et al, 2016 [94]; Castronovo et al, 2009 [77]; Chen et al, 2016 [95]; Chorianopoulos et al, 2016 [96]; Dagliati et al, 2018 [66]; Deodhar et al, 2015 [65]; Garcia-Marti et al, 2017 [97]; Gligorijevi et al, 2017 [98]; Gotz et al, 2014 [76]; Guo et al, 2007 [69]; Haque et al, 2014 [99]; Hardisty et al, 2010 [100]; Huang et al, 2015 [101]; Hund et al, 2016 [90]; Ji et al, 2012 [102]; Ji et al, 2013 [81]; Jiang et al, 2016 [103]; Jinpon et al, 2017 [83]; Kaieski et al, 2016 [104]; Katsis et al, 2017 [105]; Kostkova et al, 2014 [75]; Kruzikas et al, 2014 [106]; Lavrac et al, 2007 [70]; Lu et al, 2017 [71]; Luo et al, 2016 [78]; Maciejewski et al, 2011 [79]; Marek et al, 2015 [108]; Mitrpanont et al, 2017 [109]; Mittelstadt et al, 2014 [110]; Ozkaynak et al, 2015 [111]; Park et al, 2018 [112]; Perer et al, 2015 [113]; Proulx et al, 2006 [114]; Shaban-Nejad et al, 2017 [84]; Soulakis et al, 2015 [115]; Tate et al, 2014 [87]; Tilahun et al, 2014 [88]; Toddenroth et al, 2014 [116]; Torres et al, 2012 [117]; Widanagamaachchi et al, 2017 [72]; Xing et al, 2010 [91]; Xu et al, 2013 [73]; Yan et al, 2013 [118]; Yu et al, 2017 [82]; Yu et al, 2018 [74]
Academics and data scientists	47 (85)	Abusharekh et al, 2015 [67]; Afzal et al, 2011 [85]; Antoniou et al, 2010 [93]; Baytas et al, 2016 [80]; Bryan et al, 2015 [64]; Byrd et al, 2016 [94]; Chorianopoulos et al, 2016 [96]; Dagliati et al, 2018 [66]; Deodhar et al, 2015 [65]; Garcia-Marti et al, 2017 [97]; Gligorijevi et al, 2017 [98]; Gotz et al, 2014 [76]; Guo et al, 2007 [69]; Haque et al, 2014 [99]; Hardisty et al, 2010 [100]; Huang et al, 2015 [101]; Hund et al, 2016 [90]; Ji et al, 2012 [102]; Ji et al, 2013 [81]; Jiang et al, 2016 [103]; Jinpon et al, 2017 [83]; Kaieski et al, 2016 [104]; Katsis et al, 2017 [105]; Kostkova et al, 2014 [75]; Kruzikas et al, 2014 [106]; Lavrac et al, 2007 [70]; Lu et al, 2017 [71]; Luo et al, 2016 [78]; Maciejewski et al, 2010 [107]; Maciejewski et al, 2011 [79]; Marek et al, 2015 [108]; Mitrpanont et al, 2017 [109]; Mittelstadt et al, 2014 [110]; Ozkaynak et al, 2015 [111]; Park et al, 2018 [112]; Perer et al, 2015 [113]; Proulx et al, 2006 [114]; Tate et al, 2014 [87]; Tilahun et al, 2014 [88]; Toddenroth et al, 2014 [116]; Torres et al, 2012 [117]; Widanagamaachchi et al, 2017 [72]; Xing et al, 2010 [91]; Xu et al, 2013 [73]; Yan et al, 2013 [118]; Yu et al, 2017 [82]; Yu et al, 2018 [74]
Clinicians	21 (38)	Abusharekh et al, 2015 [67]; Alonso et al, 2012 [92]; Antoniou et al, 2010 [93]; Baytas et al, 2016 [80]; Benis et al, 2017 [89]; Bryan et al, 2015 [64]; Chorianopoulos et al, 2016 [96]; Dagliati et al, 2018 [66]; Gotz et al, 2014 [76]; Haque et al, 2014 [99]; Huang et al, 2015 [101]; Hund et al, 2016 [90]; Lu et al, 2017 [71]; Mitrpanont et al, 2017 [109]; Mittelstadt et al, 2014 [110]; Ozkaynak et al, 2015 [111]; Perer et al, 2015 [113]; Soulakis et al, 2015 [115]; Toddenroth et al, 2014 [116]; Widanagamaachchi et al, 2017 [72]; Xu et al, 2013 [73]
Policy and decision makers	7 (13)	Ji et al, 2013 [81]; Kruzikas et al, 2014 [106]; Maciejewski et al, 2011 [79]; Mitrpanont et al, 2017 [109]; Tilahun et al, 2014 [88]; Torres et al, 2012 [117]; Yu et al, 2017 [82]
Consumers and public	5 (9)	Antunes de Mendonca et al, 2015 [86]; Ji et al, 2013 [81]; Kaieski et al, 2016 [104]; Maciejewski et al, 2011 [79]; Yu et al, 2017 [82]
Industry (software, pharmaceutical, and insurance)	3 (5)	Gotz et al, 2014 [76]; Perer et al, 2015 [113]; Yu et al, 2018 [74]

### Terminology Related to Visualization and Analytics

**Figure 4:**
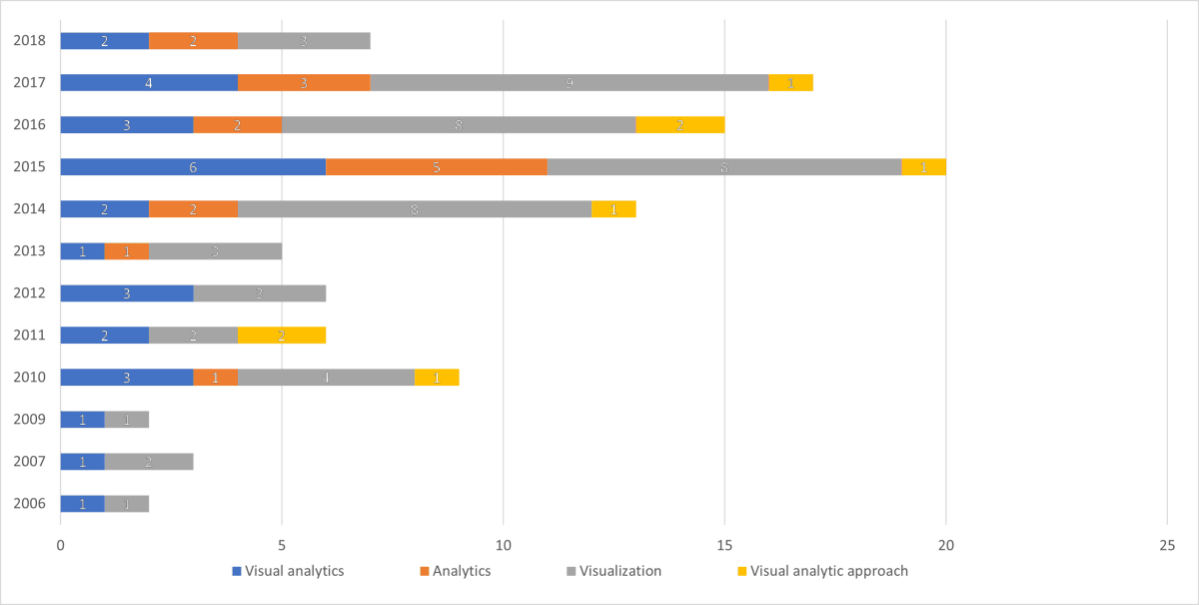
Use of terminology from January 01, 2005, to March 30, 2019.

We searched for the use of VA and its variations in the articles. Terms that indicated the use of VA included “visualization” (52/55, 95%), “visual analytics” (29/55, 53%), “analytics” (16/55, 29%), and “visual analytic approach” (8/55, 15%) as the employed method. The years when the term “visual analytics” was most commonly used were 2009 and 2017. As visualization was mentioned in the vast majority of the articles, alternative terms used for the analytic engine included data mining and ML techniques. Figure 4 displays the use of terms by year.

### Tool Name, Analytic Capability, and Goal

Related to the use of specific tools, articles that mentioned the name of the tools or base applications (34/55, 68%) are listed in Textbox 3.

In terms of analytic capability, these included tools primarily meant for descriptive analytics (52/55, 95%), exploratory analyses of complex data sets (23/55, 42%), and predictive analytics (13/55, 24%). There were no articles on prescriptive analytics. Among the overlap in the analytic capability of the tools, 3 studies (5%) mentioned descriptive and predictive analytic capabilities along with visual exploration of complex data sets [64-66]. There were 11 applications with both descriptive and predictive analytic capabilities [64-74].

We further categorized whether the application, tool, or method targeted population health and HSR decision support, knowledge discovery, or both, as its goal. There was an overlap in the application goals: decision support (44/55, 80%), knowledge discovery (35/55, 64%), or both goals (29/55, 53%).

**Name of the tool and base application (if provided).**

Abusharekh et al, 2015 [67]

- H-Drive; information analytics based on R

Ali et al, 2016 [68]

- ID-Viewer

Alonso et al, 2012 [92]

- EPIPOI based on Matlab

Antoniou et al, 2010 [93]

- dAUTObase

Antunes de Mendonca et al, 2015 [86]

- On the basis of Triplify, SQL, PHP, and SPARQL EndPoint

Baytas et al, 2016 [80]

- PhenoTree

Benis et al, 2017 [89]

- DisEpi, R-based

Bryan et al, 2015 [64]

- EpiSimS

Chen et al, 2016 [95]

- SaTScan software

Chorianopoulos et al, 2016 [96]

- Flutrack.org

Dagliati et al, 2018 [66]

- MOSAIC dashboard; data mining using R and Matlab; JavaScript; HTML; Google Charts for GUI

Deodhar et al, 2015 [65]

- EpiCaster

Haque et al, 2014 [99]

- Microsoft SQL Server’s BI tool stack and ASP.NET

Hardisty et al, 2010 [100]

- LISTA-Viz

Hund et al, 2016 [90]

- Sub-VIS; based on D3.JS2

Ji et al, 2012 [102]

- ESMOS (Epidemic sentiment monitoring system)

Ji et al, 2013 [81]

- ESMOS (Epidemic sentiment monitoring system)

Jiang et al, 2016 [103]

- Health-Terrain

Jinpon et al, 2017 [83]

- Community well-being assessment system (CWBAS)

Kaieski et al, 2016 [104]

- Vis-Health

Kostkova et al, 2014 [75]

- medi+board

Lavrac et al, 2007 [70]

- MediMap

Lu et al, 2017 [71]

- Southampton breast cancer data system (SBCDS)

Luo et al, 2016 [78]

- GS-EpiViz

Maciejewski et al, 2011 [79]

- PanViz

Marek et al, 2015 [108]

- R with spacetime, gstat and plotKML; and Google Earth

Mitrpanont et al, 2017 [109]

- SAGE2

Ozkaynak et al, 2015 [111]

- EventFlow and Discrete Time Markov Chains

Perer et al, 2015 [113]

- Care pathway explorer

Proulx et al, 2006 [114]

- nSpace and GeoTime

Shaban-Nejad et al, 2017 [84]

- Population health record (PopHR)

Tate et al, 2014 [87]

- TrialViz

Yan et al, 2013 [118]

- ISS (syndromic surveillance system)

Yu et al, 2017 [82]

- Patient-provider geographic map

Yu et al, 2018 [74]

- Watson Analytics

Afzal et al, 2011 [85]; Byrd et al, 2016 [94]; Castronovo et al, 2009 [77]; Garcia-Marti et al, 2017 [97]; Gligorijevi et al, 2017 [98]; Gotz et al, 2014 [76]; Guo et al, 2007 [69]; Huang et al, 2015 [101]; Katsis et al, 2017 [105]; Kruzikas et al, 2014 [106]; Maciejewski et al, 2010 [107]; Mittelstadt et al, 2014 [110]; Park et al, 2018 [112]; Soulakis et al, 2015 [115]; Tilahun et al, 2014 [88]; Toddenroth et al, 2014 [116]; Torres et al, 2012 [117]; Widanagamaachchi et al, 2017 [72]; Xing et al, 2010 [91]; Xu et al, 2013 [73]

- Not mentioned

Multimedia Appendix 4 [64-118] details the analytic capability and goals of the application, indicating whether the analysis was carried out for knowledge discovery or decision support, whether the article was presented as a framework for VA, and whether the methodology itself followed one or more frameworks.

### Framework Presented or Followed

A total of 24% (13/55) articles presented frameworks for VA methods, which we categorized into 7 types based on the major theories, applications, and functions that the study authors purported to use in their methods:

1. Data integration, monitoring, and management [67,68,71,75].
2. Combining querying, mining, and visualization for electronic medical records (EMRs) [76].
3. Disease mapping, hypotheses generation, clinical decision making, and knowledge discovery [66,77,78].
4. Simulation and modeling, including statistical analysis [64,79].
5. Phenotyping for a VA tool [80].
6. Social media VA [81].
7. Studying geographic variations in access to care [82].

A total of 29% (16/55) articles used a framework in their methods, which we broadly categorized into 6 types based on their application to the use case:

1. Studying access to care [83,84].
2. Analytics [78].
3. Application development [65,67,85,86].
4. Data quality, linkage, and flow [72,87,88].
5. Knowledge discovery [89].
6. Visualization [64,66,71,77,90].

Table 4 lists both kinds of frameworks and related references. The abovementioned categories are based on the objectives of the VA application, as mentioned by the authors in their studies.

**Table 4:** Articles proposing a framework and using frameworks for their visual analytics work with quoted references (if provided).

Study (reference)	Presents a framework	Uses one or more frameworks for VA^a^ work
Abusharekh et al, 2015 [67]	Health data analytics framework incorporating data management, analytics, and visualization	Portal developed using Liferay and Vaadin frameworks
Afzal et al, 2011 [85]	N/A^b^	On the basis of the recommendations by Jankun-Kelly and Ma. [119]
Ali et al, 2016 [68]	Framework for data integration and analytics with various modules related to data acquisition, cleaning, parsing and analysis	N/A
Antunes de Mendonca et al, 2015 [86]	N/A	Resource development framework for queries, with SQL and others
Baytas et al, 2016 [80]	Phenotyping framework for a VA tool	N/A
Benis et al, 2017 [89]	N/A	Knowledge discovery in databases framework [120]
Bryan et al, 2015 [64]	Presents a framework for simulating and analyzing data. Visual engine also has a built-in statistical framework based on others	On the basis of the 3 frameworks [36-138]
Castronovo et al, 2009 [77]	Conceptual framework for dynamic mapping; hypotheses generation for disease seasonality	On the basis of the Harrower principles [139]
Dagliati et al, 2018 [66]	Presents a framework as a general model for chronic disease clinical decision support and knowledge discovery	Temporal abstraction [140]
Deodhar et al, 2015 [65]	N/A	Middleware based on the Model View Controller Framework
Gotz et al, 2014 [76]	Combines 3 components, such as visual query, pattern mining, and interactive vis components, in a single framework enabling an ad hoc event sequence analysis	N/A
Hund et al, 2016 [90]	N/A	Uses the detected subspaces of the OpenSubspace Framework and Visualization follows Shneiderman [141,142]
Ji et al, 2013 [81]	Framework considers several diseases; novel 2-step sentiment classification combining clue-based searching and ML methods to first filter out the nonpersonal; identifying all personal tweets; then distinguishing personal into negative and nonnegative sentiment tweets	N/A
Jinpon et al, 2017 [83]	N/A	Community Wellbeing Framework [143]
Kostkova et al, 2014 [75]	Framework depicts processes and components required for automated data monitoring across multiple real-time data channels [P Kostkova. A roadmap to integrated digital public health surveillance: the vision and the challenges. In Proceedings of the 22nd international conference on World Wide Web (WWW '13). 687-694., 2013]	N/A
Lu et al, 2017 [71]	Process-driven framework presented, with data, functional, and user layers	Lifelines framework sits within the University Hospital Southampton Clinical Data Environment as a model for the exploratory analysis of data
Luo et al, 2016 [78]	Presents a new framework for effective disease-control strategies, starting from identifying geo-social interaction patterns. Framework further used to structure the design of a VA tool with 3 components: reorderable matrix for geo-social mixing patterns, agent-based epidemic models, and combined visualization methods	Susceptible-Exposed-Infectious-Removed agent-based modeling
Maciejewski et al, 2011 [79]	The PanViz Visualization framework uses a mathematical epidemic model to calculate population dynamics and infection rate data	N/A
Shaban-Nejad et al, 2017 [84]	N/A	Semantic population health framework introduced in the tool by using type I evidence or causal knowledge to arrange health indicators along the lines of the determinants of health framework [144]
Tate et al, 2014 [87]	N/A	Data quality framework [145]
Tilahun et al, 2014 [88]	N/A	Silk Link Discovery Framework [146]
Widanagamaachchi et al, 2017 [72]	N/A	ViSUS framework for designing dataflow [147]
Yu et al, 2017 [82]	Introduces Visualization framework to aid health care policy makers and hospital administrators to visualize, identify, and optimize the geographic variations of access to care	N/A

^a^VA: visual analytics.

^b^N/A: not applicable.

### Data Characteristics: Source, Use Cases, Structure, and Type

VA engines differ in their application, given their capability to process data from multiple data sets or various sources such as social media text data, administrative data, global repositories, and other internet sources. In the included studies, the data sources that were processed by the analytic engines varied, involving single data sources (32/55, 58%), multiple data sources (22/55, 40%), or both (6/55, 11%).

In use cases where multiple data sources were involved, there were overlaps within the categories of data sources: administrative or national survey data (17/55, 31%), EMR or EHR data (17/55, 31%), spatiotemporal data (16/55, 29%), web or social media data (8/55, 15%), and simulation data (6/55, 11%).

Articles focused on structured (40/55, 73%), unstructured (13/55, 24%), and semistructured data (5/55, 9%). The data sources were administrative data that included registry and national survey data (19/55, 35%), EMR or EHR data (17/55, 31%), spatiotemporal data (16/55, 29%), simulation data (6/55, 11%), and web or social media data (8/55, 15%). Multimedia Appendix 5 [64-118] details the source, type, and application to the use cases.

### Analytic and Visualization Engines

From the articles, we gleaned information on the analytic engine, tools, and specific methods used, such as the algorithms for the analytic methods. The tool’s analytic engine, its data processing, analysis, and subsequent data visualization varied greatly. In addition, details about the analytic and visualization engines have not been consistently reported.

We categorized the data for the type of problem that the application addressed by the major analytic techniques used for summarizing the results. There were 7 major categories: infectious disease modeling and surveillance (21/55, 38%); medical record pattern identification (20/55, 36%); population health monitoring (9/55, 16%); health system resource planning (2/55, 4%); and data manipulation, disease mapping, and sentiment analysis (1/55, 2%).

The analytic approaches undertaken included data querying (11/55, 20%); statistical modeling (11/55, 20%); clustering (9/55, 16%); natural language processing (NLP), pattern mining, classification, data mining, dimensionality reduction, predictive modeling, and other ML methods (4/55, 7%); and graph partitioning, neural networks, simulation-based predictions, and other statistical analyses (1/55, 2%).

The problems addressed and the analytic techniques used are summarized in Table 5. Multimedia Appendix 5 provides in-depth information on the data type, analytic and visual engines, and related techniques. Major tools employed for developing the applications included R-based tools (7/55, 13%); D3.JS (4/55, 7%); SQL (4/55, 7%); Java-based tools (3/55, 5%); Python-based tools, HTML 5, or Google Maps application programming interface (API; 2/55, 4%); and not reported (15/55, 27%). The 16 remaining articles mentioned the use of one of the following: Open Layers 3, OwlAPI, SaTScan, SQL and Google Maps API, IBM Watson Analytics, GeoViz Toolkit, Flutrack API, Weka, GeoTime, ESRI ArcMap, Excel2RDF and Sgvigler, C#, JFreeChart, MS Silverlight-based Pivot Viewer, Weka and Tableau, and Matlab.

**Table 5:** Problem categories and major analytic methods.

Analytic method	Categories of problems with the number of articles mentioning the use of specific analytic methods							
	Data manipulation	Disease mapping	Health system resource planning	Infectious disease modeling and surveillance	Medical record pattern identification	Population health monitoring	Sentiment analysis	Total
Data querying	1	1	—^a^	3	5	1	—	11
Statistical modeling	—	—	1	8	2	—	—	11
Clustering	—	—	—	—	7	1	1	9
Natural language processing	—	—	—	3	1	—	—	4
Other machine learning	—	—	—	1	—	3	—	4
Pattern mining	—	—	—	1	3	—	—	4
Classification	—	—	1	—	—	1	—	2
Data mining	—	—	—	—	—	2	—	2
Dimensionality reduction	—	—	—	1	1	—	—	2
Predictive modeling	—	—	—	1	1	—	—	2
Graph partitioning	—	—	—	1	—	—	—	1
Neural networks	—	—	—	1	—	—	—	1
Simulation-based predictions	—	—	—	1	—	—	—	1
Statistical analysis	—	—	—	—	—	1	—	1
Total	1	1	2	21	20	9	1	55

^a^Null values.

**Figure 5:**
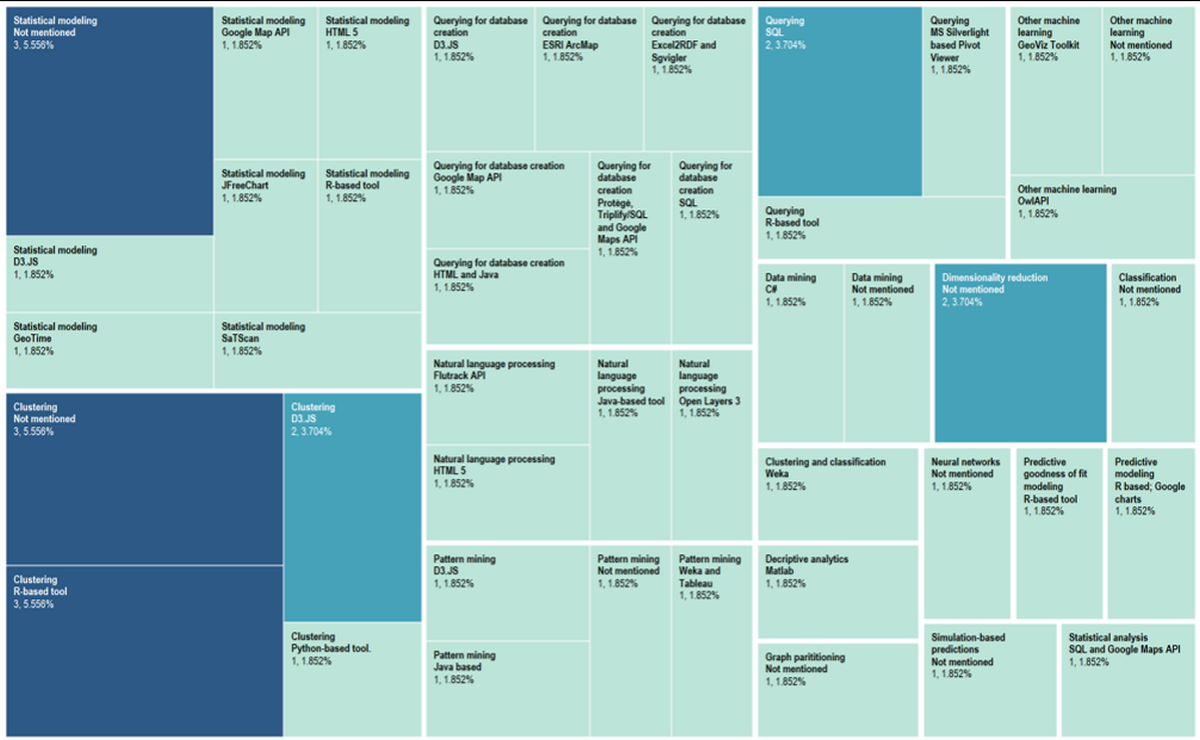
Analytic methods and proportional distribution of tools employed. API: application programming interface; MS: Microsoft; SQL: structured query language.

The distribution of the tools used according to the analytic methods is illustrated in Figure 5. Among the most often used tools were R-based tools and packages, D3.JS, and Google Maps API. Almost all articles mentioned a different combination of tools that they had used for the VA application.

Similarly, various visualizations were used to represent the analysis of the data processed by the analytic engine. Visualizations were mostly interactive, with a dashboard presenting statistics or detailed information regarding specific populations or variables of interest. The major visualization types were as follows: maps (17/55, 31%), timelines (8/55, 15%), heat maps (6/55, 11%), choropleth maps (6/55, 11%), bubble charts (3/55, 5%), flow maps (2/55, 4%), and scatterplots (2/55, 4%). The remaining 12 applications presented one of the following visuals: spatial plots, history tree view, stacks and cards, line, bar, causal diagram, cards, stacked bar, population pyramid, circular tree, ranked trees, Sankey diagram, and relationship graph.

### Domains of Health Care, Problem Category, and Related Analytic Methods

Of the articles, 98% (54/55) focused on population health, whereas 33% (18/55) focused on HSR. There was a considerable overlap, as 17 HSR articles had a population focus. Of the population health articles, 44% (24/55) were on clinical populations focusing on a condition or cluster of conditions and 31% (17/55) provided population demographics. Epidemic monitoring and modeling for certain conditions was the focus of 33% (18/55) studies, whereas 49% (27/55) were spatiotemporal health care articles.

Among the HSR articles, 27% (15/55) were on health service utilization, 18% (10/55) focused on access to care, and 4% (2/55) were related to health care costs. The details are provided in Multimedia Appendix 6 [64-118].

We further categorized the types of problems that the application addressed. The 4 major problem categories were infectious disease modeling and surveillance (21/55, 38%), medical record pattern identification (19/55, 35%), population health monitoring (9/55, 16%), and health system resource planning (2/55, 4%). One use case was for data manipulation, disease mapping, health record pattern identification, and sentiment analysis.

**Figure 6:**
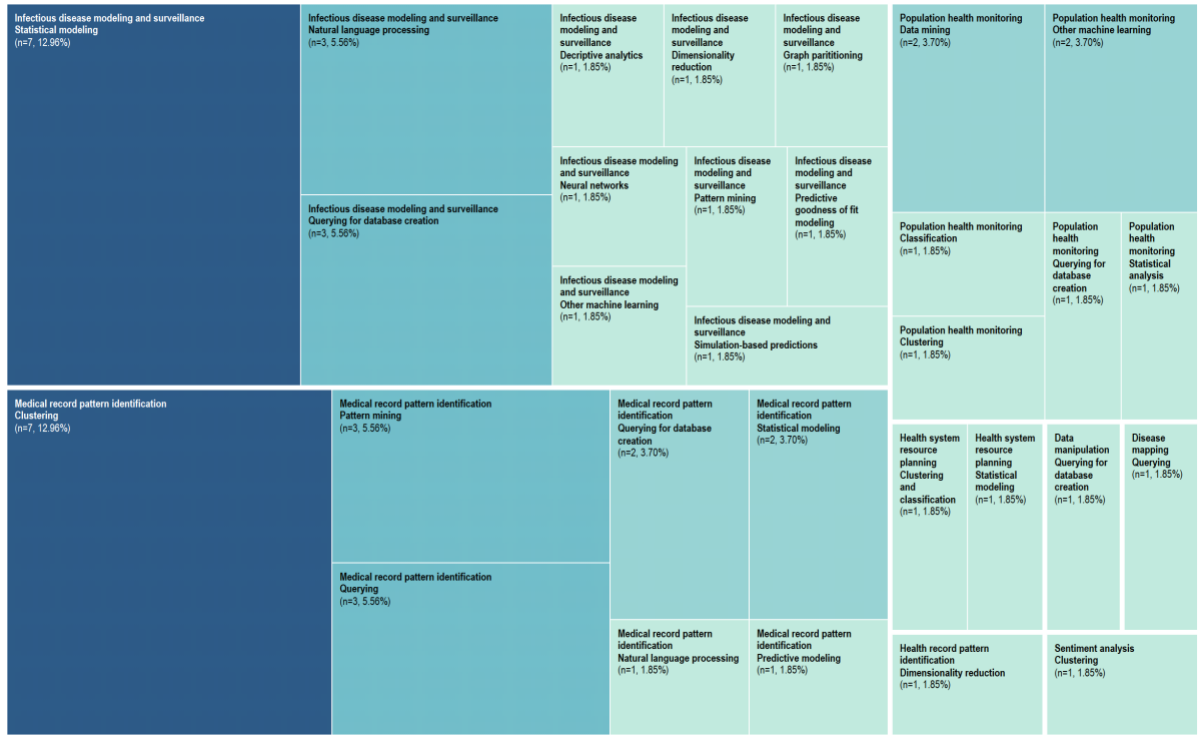
Problem categories and proportional distribution of analytic methods used.

Figure 6 details the relative distribution of the analytic methods used for the categories of problems. The color-coded tree map reveals clustering and statistical modeling as the major choice for medical record pattern identification and infectious disease modeling and surveillance, both methods comprising 13% (7/55) of all use cases. The second most common methods included NLP, querying for database creation, pattern mining, and data querying, each comprising 5% (3/55) of all methods. Other varied methods are shown in the figure to reveal the overall trends found in the use of methods according to the problem addressed by the application.

### Availability of the Tool, Uptake, and Co-Design

A total of 21 VA tools were in use at the time of publication of the original article, whereas others were either not available or prototypes. Moreover, 7 dashboards or tools were accessible for public use, whereas 13 tools were either developed using free, open source tools such as R or Weka or their source code was provided. Furthermore, 38% (21/55) articles did not mention the tools used to develop the application or the base application.

Relevant to co-design or evaluation of the tool, 10 articles involved domain experts, multidisciplinary teams, or user evaluations for the development and improvement of the VA application, whereas other articles did not mention this aspect. Relevant details have been captured in Multimedia Appendix 7 [64-118].

### Innovation and Limitations of VA Applications

All applications offered an innovative edge over others at the time of their publication. These mostly pertained to the analytic engine and techniques such as better workflow, automation, development of a framework, and use of advanced techniques such as ML. Similarly, the limitations of the applications were provided in varying detail, with 29% (16/55) articles not mentioning any limitations, as shown in Multimedia Appendix 7.

## Discussion

### Significance of the Review

The aim of this scoping review is to review the literature on VA methods, specifically their application to the fields of population health and HSR. Given the large variety, heterogeneity, and velocity of data sources, public health data belong to the category of big data [50], which are increasingly being generated and made available from administrative, EMR, and EHR sources. Examples of large population-level repositories include the United Kingdom’s Clinical Practice Research Datalink database, the largest collection of anonymized primary care patient records [87]; the Canadian administrative health data sets [121]; and the US National Health and Nutrition Examination Survey, collected since the 1970s [91].

Our scoping review summarizes VA methods applied to use cases in population health and HSR. As a multidisciplinary team, we presented the results from multiple perspectives, including those of data scientists, population health and HSR practitioners, and policy and decision makers. This is important in the wake of the COVID-19 pandemic, where multiple VA products for pandemic monitoring have surfaced for guiding the pandemic response [122,123].

We discuss the implications and contributions of this review for researchers and practitioners in the related health care areas of public and population health and HSR, expanding on aspects of specific import. We further offer targeted recommendations for defining, reporting, and leveraging the potential of VA methods and applications.

### Reporting Checklist for VA Applications

The field of visualization and analytics is extremely broad, with various applications in different health care and other sectors. We had to rely mainly on iterative screening to filter out articles that were not relevant to the study objectives, for example, articles without use cases including usability studies, evaluations, human-computer interaction, and GIS studies.

There is a need for better reporting on the details of the applications for reproducibility and transparency. This specifically relates to the tool’s capability, application beyond the use case, target audience, study objectives, and study settings. In many articles, we found the statistical and analytic methods lacking in detail, in particular on the tools used for the analysis, the algorithms tested and applied, and the reasons for choosing one particular analysis over the other.

Similarly, some articles from proprietary or prototypical tools did not offer any detail on the analytic engine, while only discussing the functional aspects of the application. Many articles did not elaborate on how visualization presentations should be interpreted. Most articles did not provide reasons and processes for the selection of the visualization, its strength over others, and how the interactive functions could offer more insight.

Such details would help situate the literature and resultantly be useful for better reproducibility, development, and adaptability of prototypical and established methods to different scenarios.

Towards this goal, we developed a standard reporting checklist (Multimedia Appendix 8) for reporting VA methods, particularly visual and analytics engines, as is the practice for reporting research methods such as statistical techniques [124], and qualitative and quantitative studies [125,126]. As mentioned previously, 4 recent systematic reviews covered areas of VA applications in health [8,17-19]. Although these reviews offered excellent summaries from different areas of health care and informatics, we found that there was no reporting standard followed, indicating the need for such a checklist. We further sought recent literature on COVID-19–related VA products. One of the most known COVID-19–related products is the web-based dashboard for country-level data by Johns Hopkins University [123]. Although the experts involved have not yet published a paper detailing the methods for its development, a high-level correspondence article was published in the reputed journal *Lancet* [123]. In this article, the authors cite the issues and process of developing a data stream for the dashboard. In contrast, the authors of another VA product mapping the COVID-19–related mobility pattern changes in US counties detail the methods, features of the web-based platform, data sources, system design, and insights from the results in their publication [122].

On the basis of the findings from these papers and those included in the scoping review, we have proposed a checklist for reporting VA applications (Multimedia Appendix 8) to fulfill the need for standard reporting aimed at optimizing productivity from research efforts [3].

### Proposed Definition for VA in Health Care

We adhered to the definition for VA applications in health care by Ola and Sediq [50] for which both an analytic and visual engine must be included. However, we found that despite reporting analytic techniques, including an analytic engine, many articles did not state it as such. Although “visualization” as a term was mentioned in all articles, analytic techniques were not mainly classified as analytic engines. This could be due to the different use of language and understanding within the data science communities of practice. Hence, VA as a term with a technical definition does not seem firmly established, at least in the health care literature. This can also be seen in various authors’ work where they borrow from the original definition of VA by Thomas and Cook [2] being “*the science of analytical reasoning facilitated by interactive visual interfaces.”*

Thomas and Cook [2] define VA as “the science of analytical reasoning facilitated by interactive visual interfaces,” whereas Keim et al [3] extended the concept to “automated analysis techniques with interactive visualizations for an effective understanding, reasoning and decision making on the basis of very large and complex data sets.” Borrowing from the seminal works of Ola and Sedig [50], Keim et al [3], and Thomas and Cook [2], we recommend using the following adapted definition of VA, especially in areas related to public and population health: “an approach, method or application for analytic reasoning, exploration, knowledge discovery, and sense making of complex data, through the use of one or more interactive visual interfaces, employing analytic and visual engines.” In our definition, we keep the aim of the VA technique at the fore to provide context to the method, while expanding on the limited concept of VA to *computational tools* [3,50]. We emphasize the analytic and visual engines to help delineate the methods from other fields, such as visualization. We also emphasize it as it helps to define and report the methods better, for which we included a checklist for reporting (Multimedia Appendix 8).

### VA Methods, Frameworks, and Tools

We followed a broad definition of frameworks to summarize the VA methods in developing the applications. Although presenting the detailed findings from these frameworks is beyond the scope of this review, we broadly categorized their types, as it can be valuable to learn from the conceptual and theoretical bases of this innovative method. Studying both types of frameworks helps situate the methods for adaptation by researchers and practitioners. Among the variety of VA frameworks presented, most were related to disease mapping and for knowledge discovery and hypothesis generation [66,77,78]. This is consistent with the findings of the goals and analytic capabilities of the tools that we summarize. Although there is a trend toward the application of ML methods to EMR data sets, we found 1 framework for mining and visualizing trends and patterns from these data sets [76].

The majority of the applications were prototypes, with only 5 in use at the time of publication. In part, this may be due to publication bias of newer VA techniques. Studies using proprietary tools known for their visualization engines, such as Tableau [148], Qlik [149], and Power BI [150], were exceptionally uncommon in the articles that were reviewed. Hence, future research may seek to survey the population health and HSR practitioners to gain an understanding of the VA tools that are part of their daily decision-making processes and reasons for not publishing their findings and experiences.

### Settings, Target Audience, and Co-Design Initiatives

As we limited our search to English language articles, the use cases from the included studies were mostly from the United States, with fewer studies from Europe or other countries. Most of the authors and use cases were from the same country, aside from one exception, an author from Canada working on a use case from the United States [91].

As expected, population health and HSR practitioners were the most common intended target audience, followed by academic researchers and clinicians. Policy and decision makers as well as the general public were not the main target audiences. Although VA is related to data visualization and is being increasingly employed to convey insights from the data, we contend that the use of VA is still in the developmental stages. This corresponds to our finding that most applications were prototypes.

There were 5 studies that were aimed at consumers, whereas 7 studies targeted policy and decision makers. However, as participatory approaches are being emphasized for better uptake and development of creative solutions [127], a concern related to co-designing applications was the lack of involvement of stakeholders, such as decision makers, and patient groups. It is important to note that the lack of participatory co-design approaches in developing applications could be one reason for the overall finding of slow uptake of these methods in population health and HSR.

### Trends and Potential for VA Applications

As the results show, the use of VA varies greatly. In addition, due to inconsistent reporting of the settings and target audiences in the included papers, we made a calculated judgment on the trends in the use of these techniques. As most of the studies were conducted in academic circles, we infer that these methods are still in development in the population health and HSR communities of practice. Hence, the uptake of these methods has been slow in these interrelated areas of health care. This is not unexpected, as the field has been termed nascent, while the application of newer techniques in public health has been rather delayed [50].

As most tools focused on descriptive analytics, with about half aimed at visual exploration of complex data sets, the trend in the use of these methods toward knowledge discovery and decision support is notable. This could be due to the availability of increasing and expanded data sets from EMR systems. For ML, clustering, classification, and NLP are methods of choice for structured and text-based data sets. Many population health applications are related to mapping, spatiotemporal distribution, and modeling for diseases and disease control. In HSR, few articles dealt with issues of access, utilization, and costs of services.

Most problems addressed related to infectious disease epidemiology, with clustering and statistical modeling being the most commonly used analytic methods. The articles mentioned a unique tool, a combination of tools, or did not mention the tool or base application, which made it difficult to summarize the types of tools used. However, as shown in Figure 5, R-based tools, Google Maps API and D3.JS, as well as a variety of other tools were used for the VA applications.

In addition, there is added value in using VA to obtain and combine multiple data sources to construct a fuller picture toward the question of inquiry. As our results show, the analytic engine in most use cases combines multiple data sources, such as EMRs, to social media sources. As Keim et al [3] point out, VA can contribute to solving various complex problems in sectors including engineering, financial analysis, environment and climate change, and socio-economic conditions. Socio-economic considerations in health, known as the social determinants of health, are being increasingly researched in the context of accessibility, health, and overall quality of life of populations [128]. In addition, VA has the potential to address the varied shared application problems in health and related sectors at an abstract level [3].

### Learning Health Systems and COVID-19–Related VA Products

Learning health systems are geared toward continuous evidence-based quality improvement [129]. There are multiple challenges in building such systems that generate knowledge and insights on proposed improvements [130]. In the wider context, this review allows fellow researchers, practitioners, and decision makers to appreciate the potential presented by VA techniques in meeting challenges in operationalizing and building automated data-driven learning health systems [131]. VA techniques have the ability for sense making and leveraging big data from multiple sources to operationalize such learning health systems [33,132].

As has been the case in the last few months of the COVID-19 crisis, a plethora of VA products have surfaced, aimed at clinical practitioners, population health and health service researchers, policy makers, and the general public [122,123,133]. Such VA products are being increasingly sought for epidemiologic surveillance, monitoring, and planning of health services, in addition to apprising the public on the magnitude of the pandemic. It will be especially useful for research replicability and transparency to describe the development and features of such products in sufficient detail, toward which we presented a reporting checklist (Multimedia Appendix 8), for aspects that we found to be important in reporting methods and functionality of an application. We are confident that this will serve novice and expert researchers alike as a reminder to showcase the depth and breadth of their efforts in developing a unique application.

### Limitations

We based our inquiry of VA methods on information from peer-reviewed journal articles and full conference papers. We did not include book chapters, theses, short papers, editorials, non–peer-reviewed reports, conference abstracts, and live websites using VA techniques. We limited our review to the year 2005 onward, and we did not explore subject-specific databases from mathematics, geography, and computer sciences. We sought to limit our findings to proposed or established methods that have been either published or presented and applied to actual use cases. We included full conference papers in the review, but many conferences do not publish proceedings, such as the annual Tableau conference and the Health Analytics Summit. Use cases discussed at these meetings mostly involve front-end proprietary tools. Hence, the complete spectrum of the use of such tools could not be covered in this review. However, we followed the highest methodological standards for conducting systematic reviews. This included developing a multidisciplinary team of health researchers and data scientists, following established review frameworks with at least two independent reviewers at each step, and being guided by a dedicated information specialist. Our search strategy was developed over multiple iterations and was peer reviewed using the PRESS guidelines [134] by an independent third-party information specialist, whereas we published the review protocol in advance [21].

### Conclusions

VA as an innovative field holds great potential in yielding insights from big health care data, especially in the related fields of population health and HSR. This is especially relevant in the backdrop of the COVID-19 pandemic, where multiple VA products have taken center stage.

This scoping review provides a foundational understanding of the current landscape on the application of VA methods in areas of population health and HSR. We present the major VA tools, techniques, and frameworks since 2005 published in peer-reviewed papers. VA is an innovative, rapidly expanding field with its roots in many disciplines, and it is being used to build learning health systems for improving patient care, increasing access to services, controlling costs, and appropriately allocating resources [33]. It is expected that the next generation of EMR systems will leverage advanced analytics to meet the needs of diverse audiences [135]. Such systems are aimed at harmonizing patient records; creating a seamless picture of access to care at primary, secondary, and tertiary levels; and managing disease outbreaks at the population level. We also present an expanded definition for VA applications in health care, with a reporting checklist to help researchers provide solutions for greater replicability.

## Appendix

Multimedia Appendix 1 PRISMA-ScR (Preferred Reporting Items for Systematic Reviews and Meta-Analyses Extension for Scoping Reviews) Checklist for reporting scoping reviews.

Multimedia Appendix 2 MEDLINE search strategy.

Multimedia Appendix 3 Study characteristics, problem analyzed, settings, and target audience.

Multimedia Appendix 4 Analytic capability and goals.

Multimedia Appendix 5 Data types and analytic and visual engines.

Multimedia Appendix 6 Domains of health care.

Multimedia Appendix 7 Key findings, impact, innovation, availability, and limitations.

Multimedia Appendix 8 Checklist for reporting Visual Analytic applications in population health and health services research.

## Abbreviations

API: application programming interface
EHR: electronic health record
EMR: electronic medical record
GIS: geographic information system
HSR: health services research
MeSH: Medical Subject Headings
ML: machine learning
NLP: natural language processing
PRESS: Peer Review of Electronic Search Strategies
PRISMA-ScR: Preferred Reporting Items for Systematic Reviews and Meta-Analyses Extension for Scoping Reviews
VA: visual analytics

## References

[1] Wong PC, Thomas J (2004). Visual analytics. IEEE Computer Graphics and Applications.

[2] Thomas K, Cook K (2011). Illuminating the Path: the R&d Agenda for Visual Analytics.

[3] Keim D, Andrienko G, Fekete J, Carsten G, Melan G, Keim D (2008). Visual analytics: definition, process and challenges. InfoVis.

[4] Keim D, Zhang L (2011). Solving Problems With Visual Analytics. Proceedings of the 11th International Conference on Knowledge Management and Knowledge Technologies.

[5] Andrienko G, Andrienko N, Demsar U, Dransch D, Dykes J, Fabrikant SI, Jern M, Kraak M, Schumann H, Tominski C (2010). Space, time and visual analytics. Int J Geog Inf Sci.

[6] Caban JJ, Gotz D (2015). Visual analytics in healthcare--opportunities and research challenges. J Am Med Inform Assoc.

[7] Simpao AF, Ahumada LM, Gálvez JA, Rehman MA (2014). A review of analytics and clinical informatics in health care. J Med Syst.

[8] Chung Y, Bagheri N, Salinas-Perez JA, Smurthwaite K, Walsh E, Furst M, Rosenberg S, Salvador-Carulla L (2020). Role of visual analytics in supporting mental healthcare systems research and policy: a systematic scoping review. Int J Inf Manag.

[9] Kindig D, Stoddart G (2003). What is population health?. Am J Public Health.

[10] Kindig D (2007). Understanding population health terminology. Milbank Q.

[11] (2019). Health Services Research. Canadian Institutes of Health Research.

[12] Lohr KN, Steinwachs DM (2002). Health services research: an evolving definition of the field. Health Serv Res.

[13] Martin-Sanchez F, Verspoor K (2018). Big data in medicine is driving big changes. Yearb Med Inform.

[14] Raghupathi W, Raghupathi V (2014). Big data analytics in healthcare: promise and potential. Health Inf Sci Syst.

[15] White SE (2014). A review of big data in health care: challenges and opportunities. OAB.

[16] Simpao AF, Ahumada LM, Rehman MA (2015). Big data and visual analytics in anaesthesia and health care. Br J Anaesth.

[17] Islam MS, Hasan MM, Wang X, Germack HD, Noor-E-Alam M (2018). A systematic review on healthcare analytics: application and theoretical perspective of data mining. Healthcare (Basel).

[18] West VL, Borland D, Hammond WE (2015). Innovative information visualization of electronic health record data: a systematic review. J Am Med Inform Assoc.

[19] Wu D, Chen A, Manning J, Levy-Fix G, Backonja U, Borland D, Caban JJ, Dowding DW, Hochheiser H, Kagan V, Kandaswamy S, Kumar M, Nunez A, Pan E, Gotz D (2019). Evaluating visual analytics for health informatics applications: a systematic review from the American Medical Informatics Association Visual Analytics Working Group Task Force on Evaluation. J Am Med Inform Assoc.

[20] Braunstein ML (2015). Practitioner's Guide to Health Informatics.

[21] Chishtie JA, Babineau J, Bielska IA, Cepoiu-Martin M, Irvine M, Koval A, Marchand J, Turcotte L, Jeji T, Jaglal S (2019). Visual analytic tools and techniques in population health and health services research: protocol for a scoping review. JMIR Res Protoc.

[22] Peters MD, Godfrey CM, McInerney P, Soares CB, Khalil H, Parker D (2015). Joanna Briggs Institute Reviewers' Manual: 2015 Edition.

[23] Arksey H, O'Malley L (2005). Scoping studies: towards a methodological framework. Int J Soc Res Method.

[24] Levac D, Colquhoun H, O'Brien KK (2010). Scoping studies: advancing the methodology. Implement Sci.

[25] Peters MD, Godfrey CM, Khalil H, McInerney P, Parker D, Soares CB (2015). Guidance for conducting systematic scoping reviews. Int J Evid Based Healthc.

[26] Tricco AC, Lillie E, Zarin W, O'Brien KK, Colquhoun H, Levac D, Moher D, Peters MD, Horsley T, Weeks L, Hempel S, Akl EA, Chang C, McGowan J, Stewart L, Hartling L, Aldcroft A, Wilson MG, Garritty C, Lewin S, Godfrey CM, Macdonald MT, Langlois EV, Soares-Weiser K, Moriarty J, Clifford T, Tunçalp O, Straus SE (2018). PRISMA extension for scoping reviews (PRISMA-SCR): checklist and explanation. Ann Intern Med.

[27] Cleo G, Scott AM, Islam F, Julien B, Beller E (2019). Usability and acceptability of four systematic review automation software packages: a mixed method design. Syst Rev.

[28] Shneiderman B, Plaisant C, Hesse BW (2013). Improving healthcare with interactive visualization. Computer.

[29] Viju Raghupathi WR (2013). An overview of health analytics. J Health Med Informat.

[30] Lin R, Ye Z, Wang H, Wu B (2018). Chronic diseases and health monitoring big data: a survey. IEEE Rev Biomed Eng.

[31] Liao H, Tang M, Luo L, Li C, Chiclana F, Zeng X (2018). A bibliometric analysis and visualization of medical big data research. Sustainability.

[32] Khalifa M (2018). Health analytics types, functions and levels: a review of literature. Stud Health Technol Inform.

[33] Fihn SD, Francis J, Clancy C, Nielson C, Nelson K, Rumsfeld J, Cullen T, Bates J, Graham GL (2014). Insights from advanced analytics at the veterans health administration. Health Aff (Millwood).

[34] Docherty SL, Vorderstrasse A, Brandon D, Johnson C (2017). Visualization of multidimensional data in nursing science. West J Nurs Res.

[35] Backonja U, Chi N, Choi Y, Hall AK, Le T, Kang Y, Demiris G (2016). Visualization approaches to support healthy aging: a systematic review. J Innov Health Inform.

[36] (2020). What is Public Health?. CDC Foundation.

[37] Niedźwiedzka B, Czabanowska K, Śmietana R (2008). Controlled vocabulary in public health. An overview of the achievements to date. J Public Health.

[38] Population Health. National Center for Biotechnology Information.

[39] (2007). UK: The Health Agency, Health Development List. National Public Health Language.

[40] Etches V, Frank J, di Ruggiero E, Manuel D (2006). Measuring population health: a review of indicators. Annu Rev Public Health.

[41] Cohen D, Huynh T, Sebold A, Harvey J, Neudorf C, Brown A (2014). The population health approach: a qualitative study of conceptual and operational definitions for leaders in Canadian healthcare. SAGE Open Med.

[42] Fone D, Hollinghurst S, Temple M, Round A, Lester N, Weightman A, Roberts K, Coyle E, Bevan G, Palmer S (2003). Systematic review of the use and value of computer simulation modelling in population health and health care delivery. J Public Health Med.

[43] Singh SR (2014). Public health spending and population health: a systematic review. Am J Prev Med.

[44] Casey JA, Schwartz BS, Stewart WF, Adler NE (2016). Using electronic health records for population health research: a review of methods and applications. Annu Rev Public Health.

[45] Harris JK, Beatty KE, Barbero C, Howard AF, Cheskin RA, Shapiro RM, Mays GP (2012). Methods in public health services and systems research: a systematic review. Am J Prev Med.

[46] Thompson DS, Fazio X, Kustra E, Patrick L, Stanley D (2016). Scoping review of complexity theory in health services research. BMC Health Serv Res.

[47] Gulley SP, Rasch EK, Bethell CD, Carle AC, Druss BG, Houtrow AJ, Reichard A, Chan L (2018). At the intersection of chronic disease, disability and health services research: a scoping literature review. Disabil Health J.

[48] Rowland M, Peterson-Besse J, Dobbertin K, Walsh ES, Horner-Johnson W, Expert Panel on Disability and Health Disparities (2014). Health outcome disparities among subgroups of people with disabilities: a scoping review. Disabil Health J.

[49] (2018). Finding and Retrieving HSR: Tools and Databases. US National Library of Medicine.

[50] Ola O, Sedig K (2014). The challenge of big data in public health: an opportunity for visual analytics. Online J Public Health Inform.

[51] Davenport T, Kalakota R (2019). The potential for artificial intelligence in healthcare. Future Healthc J.

[52] Pike WA, Stasko J, Chang R, O'Connell TA (2009). The science of interaction. Inf Vis.

[53] Basole R, Braunstein M, Kumar V, Park H, Kahng M, Chau DH, Tamersoy A, Hirsh DA, Serban N, Bost J, Lesnick B, Schissel BL, Thompson M (2015). Understanding variations in pediatric asthma care processes in the emergency department using visual analytics. J Am Med Inform Assoc.

[54] Basole R, Park H, Gupta M, Braunstein M, Chau D, Thompson M (2015). A Visual Analytics Approach to Understanding Care Process Variation and Conformance. Proc 2015 Workshop Visual Analytical Healthcare.

[55] Falster MO, Jorm LR, Leyland AH (2016). Visualising linked health data to explore health events around preventable hospitalisations in NSW Australia. BMJ Open.

[56] Hosseinpoor AR, Schlotheuber A, Nambiar D, Ross Z (2018). Health equity assessment toolkit plus (HEAT Plus): software for exploring and comparing health inequalities using uploaded datasets. Glob Health Action.

[57] Huang C, Syed-Abdul S, Jian W, Iqbal U, Nguyen P, Lee P, Lin S, Hsu W, Wu M, Wang C, Ma K, Li Y (2015). A novel tool for visualizing chronic kidney disease associated polymorbidity: a 13-year cohort study in Taiwan. J Am Med Inform Assoc.

[58] Martinez R, Ordunez P, Soliz PN, Ballesteros MF (2016). Data visualisation in surveillance for injury prevention and control: conceptual bases and case studies. Inj Prev.

[59] Ratwani RM, Fong A (2015). 'Connecting the dots': leveraging visual analytics to make sense of patient safety event reports. J Am Med Inform Assoc.

[60] Grant C, Osanloo A (2014). Understanding, selecting, and integrating a theoretical framework in dissertation research: creating the blueprint for your 'house'. Admin Int J.

[61] Glasgow RE, Emmons KM (2007). How can we increase translation of research into practice? Types of evidence needed. Annu Rev Public Health.

[62] Ward ME, de Brún A, Beirne D, Conway C, Cunningham U, English A, Fitzsimons J, Furlong E, Kane Y, Kelly A, McDonnell S, McGinley S, Monaghan B, Myler A, Nolan E, O'Donovan R, O'Shea M, Shuhaiber A, McAuliffe E (2018). Using co-design to develop a collective leadership intervention for healthcare teams to improve safety culture. Int J Environ Res Public Health.

[63] (2019). Jawad Chishtie. Tableau Public: Free Data Visualization Software.

[64] Bryan C, Wu X, Mniszewski S, Ma K (2015). Integrating Predictive Analytics Into a Spatiotemporal Epidemic Simulation. IEEE Conference on Visual Analytics Science and Technology.

[65] Deodhar S, Bisset K, Chen J, Barrett C, Wilson M, Marathe M (2015). EpiCaster: an integrated web application for situation assessment and forecasting of global epidemics. ACM BCB.

[66] Dagliati A, Sacchi L, Tibollo V, Cogni G, Teliti M, Martinez-Millana A, Traver V, Segagni D, Posada J, Ottaviano M, Fico G, Arredondo MT, De Cata P, Chiovato L, Bellazzi R (2018). A dashboard-based system for supporting diabetes care. J Am Med Inform Assoc.

[67] Abusharekh A, Stewart S, Hashemian N, Abidi S (2015). H-DRIVE: A Big Health Data Analytics Platform for Evidence-Informed Decision Making. IEEE International Congress on Big Data.

[68] Ali M, Ahsan Z, Amin M, Latif S, Ayyaz A, Ayyaz M (2016). ID-Viewer: a visual analytics architecture for infectious diseases surveillance and response management in Pakistan. Public Health.

[69] Guo D (2007). Visual analytics of spatial interaction patterns for pandemic decision support. Int J Geog Inform Sci.

[70] Lavrac N, Bohanec M, Pur A, Cestnik B, Debeljak M, Kobler A (2007). Data mining and visualization for decision support and modeling of public health-care resources. J Biomed Inform.

[71] Lu J, Hales A, Rew D (2017). Modelling of Cancer Patient Records: A Structured Approach to Data Mining and Visual Analytics.

[72] Widanagamaachchi W, Livnat Y, Bremer P, Duvall S, Pascucci V (2017). Interactive visualization and exploration of patient progression in a hospital setting. AMIA Annu Symp Proc.

[73] Xu S, Jewell B, Steed C, Schryver J (2013). A New Collaborative Tool for Visually Understanding National Health Indicators. Proceedings of the International Conference on Applied Human Factors and Ergonomics 2.

[74] Yu N, Zheng M, Andrade X, Patane R (2018). Visual Analysis for Exploring the Relation Between Air Pollution, Environmental Factors and Respiratory Diseases. 11th EAI International Conference on Mobile Multimedia Communications.

[75] Kostkova P, Garbin S, Moser J, Pan W (2014). Integration and Visualization Public Health Dashboard: the Medi+board Pilot Project. International World Wide Web Conference.

[76] Gotz D, Wang F, Perer A (2014). A methodology for interactive mining and visual analysis of clinical event patterns using electronic health record data. J Biomed Inform.

[77] Castronovo DA, Chui KK, Naumova EN (2009). Dynamic maps: a visual-analytic methodology for exploring spatio-temporal disease patterns. Environ Health.

[78] Luo W (2016). Visual analytics of geo-social interaction patterns for epidemic control. Int J Health Geogr.

[79] Maciejewski R, Livengood P, Rudolph S, Collins TF, Ebert DS, Brigantic RT, Corley CD, Muller GA, Sanders SW (2011). A pandemic influenza modeling and visualization tool. J Vis Lang Comput.

[80] Baytas IM, Lin K, Wang F, Jain AK, Zhou J (2016). PhenoTree: interactive visual analytics for hierarchical phenotyping from large-scale electronic health records. IEEE Trans Multimedia.

[81] Ji X, Chun SA, Geller J (2013). Monitoring Public Health Concerns Using Twitter Sentiment Classifications. IEEE International Conference on Healthcare Informatics.

[82] Yu Z, Pepe K, Rust G, Ramirez-Marquez J, Zhang S, Bonnet B (2017). Patient-provider Geographic Map: an Interactive Visualization Tool of Patients' Selection of Health Care Providers. IEEE Workshop on Visual Analytics in Healthcare.

[83] Jinpon P, Jaroensutasinee M, Jaroensutasinee K (2017). Integrated information visualization to support decision-making in order to strengthen communities: Design and usability evaluation. Inform Health Soc Care.

[84] Shaban-Nejad A, Lavigne M, Okhmatovskaia A, Buckeridge DL (2017). PopHR: a knowledge-based platform to support integration, analysis, and visualization of population health data. Ann N Y Acad Sci.

[85] Afzal S, Maciejewski R, Ebert D (2011). Visual analytics decision support environment for epidemic modeling and response evaluation. 2011 IEEE Conference on Visual Analytics Science and Technology.

[86] de Mendonça PG, Maciel C, Viterbo J (2015). Visualizing Aedes aegypti infestation in urban areas: A case study on open government data mashups. Int Pol.

[87] Tate AR, Beloff N, Al-Radwan B, Wickson J, Puri S, Williams T, Van Staa T, Bleach A (2014). Exploiting the potential of large databases of electronic health records for research using rapid search algorithms and an intuitive query interface. J Am Med Inform Assoc.

[88] Tilahun B, Kauppinen T, Keßler C, Fritz F (2014). Design and development of a linked open data-based health information representation and visualization system: potentials and preliminary evaluation. JMIR Med Inform.

[89] Benis A, Hoshen M (2017). DisEpi: compact visualization as a tool for applied epidemiological research. Stud Health Technol Inform.

[90] Hund M, Böhm D, Sturm W, Sedlmair M, Schreck T, Ullrich T, Keim DA, Majnaric L, Holzinger A (2016). Visual analytics for concept exploration in subspaces of patient groups: making sense of complex datasets with the doctor-in-the-loop. Brain Inform.

[91] Xing Z, Pei J (2010). Exploring disease association from the NHANES data: data mining, pattern summarization, and visual analytics. Int J Data Warehous Min.

[92] Alonso WJ, McCormick BJ (2012). EPIPOI: a user-friendly analytical tool for the extraction and visualization of temporal parameters from epidemiological time series. BMC Public Health.

[93] Antoniou D, Georgitsi M, Gkantouna V, Patrinos G, Poulas K, Tsakalidis A, Tzimas GE, Viennas ES (2010). Dautobase: Mining Gems on Autoimmune Diseases Utilizing Web Visualization Technologies. Proceedings of the 10th IEEE International Conference on Information Technology and Applications in Biomedicine.

[94] Byrd K, Mansurov A, Baysal O, editors (2016). Mining Twitter Data for Influenza Detection and Surveillance. Proceedings of the International Workshop on Software Engineering in Healthcare Systems.

[95] Chen C, Teng Y, Lin B, Fan I, Chan T (2016). Online platform for applying space-time scan statistics for prospectively detecting emerging hot spots of dengue fever. Int J Health Geogr.

[96] Chorianopoulos K, Talvis K (2016). Flutrack.org: open-source and linked data for epidemiology. Health Informatics J.

[97] Garcia-Martí I, Zurita-Milla R, Swart A, van den Wijngaard KC, van Vliet AJ, Bennema S, Harms M (2016). Identifying environmental and human factors associated with tick bites using volunteered reports and frequent pattern mining. Trans in GIS.

[98] Frtuni GM, Puflovi D, Stevanoska E, Jevtovi ST, Velinov G, Stoimenov L (2017). Interactive Map Visualization System Based on Integrated Semi-structured and Structured Healthcare Data. Data Integration in the Life Sciences.

[99] Haque W, Urquhart B, Berg E, Dhanoa R (2014). Using business intelligence to analyze and share health system infrastructure data in a rural health authority. JMIR Med Inform.

[100] Hardisty F, Klippel A (2010). Analysing spatio-temporal autocorrelation with LISTA-Viz. Int J Geograp Inf Sci.

[101] Huang C, Lu R, Iqbal U, Lin S, Nguyen PA, Yang H, Wang C, Li J, Ma K, Li YJ, Jian W (2015). A richly interactive exploratory data analysis and visualization tool using electronic medical records. BMC Med Inform Decis Mak.

[102] Ji X, Chun S, Geller J (2012). Epidemic Outbreak and Spread Detection System Based on Twitter Data. Health Information Science.

[103] Shenhui J, Shiaofen F, Bloomquist S, Keiper J, Palakal M, Yuni X (2016). Healthcare Data Visualization: Geospatial and Temporal Integration. 11th Joint Conference on Computer Vision, Imaging and Computer Graphics Theory and Applications - Volume 2: IVAPP.

[104] Kaieski N, Oliveira L, Villamil M (2016). Vis-Health: Exploratory Analysis and Visualization of Dengue Cases in Brazil. IEEE.

[105] Katsis Y, Balac N, Chapman D, Kapoor M, Block J, Griswold W (2017). Big Data Techniques for Public Health: A Case Study. International Conference on Connected Health: Applications, Systems and Engineering Technologies (CHASE).

[106] Kruzikas D, Higashi M, Edgar M, Macal C, Graziano D, North M (2014). Using Agent-Based Modeling to Inform Regional Health Care System Investment and Planning. 2014 International Conference on Computational Science and Computational Intelligence.

[107] Maciejewski R, Drake T, Rudolph S, Malik A, Ebert D, editors (2010). Data Aggregation and Analysis for Cancer Statistics - A Visual Analytics Approach. 43rd Hawaii International Conference on System Sciences.

[108] Marek L, Tuček P, Pászto Vít (2015). Using geovisual analytics in Google Earth to understand disease distribution: a case study of campylobacteriosis in the Czech Republic (2008-2012). Int J Health Geogr.

[109] Mitrpanont J, Roungsuriyaviboon J, Sathapornwatanakul T, Sawangphol W, Kobayashi D, Haga J, editors (2017). Extending MedThaiVis-Thai medical research visualization to SAGE2 display walls. 22nd International Conference on Information Technology.

[110] Mittelstadt S, Hao M, Dayal U, Hsu M, Terdiman J, Keim D (2014). Advanced Visual Analytics Interfaces for Adverse Drug Event Detection. Proceedings of the 2014 International Working Conference on Advanced Visual Interfaces.

[111] Ozkaynak M, Dziadkowiec O, Mistry R, Callahan T, He Z, Deakyne S, Tham E (2015). Characterizing workflow for pediatric asthma patients in emergency departments using electronic health records. J Biomed Inform.

[112] Park A, Conway M, Chen AT (2018). Examining thematic similarity, difference, and membership in three online mental health communities from reddit: a text mining and visualization approach. Comput Human Behav.

[113] Perer A, Wang F, Hu J (2015). Mining and exploring care pathways from electronic medical records with visual analytics. J Biomed Inform.

[114] Proulx P, Tandon S, Bodnar A, Schroh D, Harper R, Wright W (2006). Avian Flu Case Study with nSpace and GeoTime. IEEE Symposium On Visual Analytics Science And Technology.

[115] Soulakis ND, Carson MB, Lee YJ, Schneider DH, Skeehan CT, Scholtens DM (2015). Visualizing collaborative electronic health record usage for hospitalized patients with heart failure. J Am Med Inform Assoc.

[116] Toddenroth D, Ganslandt T, Castellanos I, Prokosch H, Bürkle T (2014). Employing heat maps to mine associations in structured routine care data. Artif Intell Med.

[117] Torres S, Eicher-Miller H, Boushey C, Ebert D, Maciejewski R (2012). Applied Visual Analytics for Exploring the National Health and Nutrition Examination Survey. 45th Hawaii International Conference on System Sciences.

[118] Yan W, Palm L, Lu X, Nie S, Xu B, Zhao Q, Tao T, Cheng L, Tan L, Dong H, Diwan VK (2013). ISS--an electronic syndromic surveillance system for infectious disease in rural China. PLoS One.

[119] Jankun-Kelly TJ, Ma KL, Gertz M (2007). A model and framework for visualization exploration. IEEE Trans Vis Comput Graph.

[120] Fayyad U, Piatetsky-Shapiro G, Smyth P (1996). The KDD process for extracting useful knowledge from volumes of data. Commun ACM.

[121] Tu K, Mitiku TF, Ivers NM, Guo H, Lu H, Jaakkimainen L, Kavanagh DG, Lee DS, Tu JV (2014). Evaluation of electronic medical record administrative data linked database (EMRALD). Am J Manag Care.

[122] Gao S, Rao J, Kang Y, Liang Y, Kruse J (2020). Mapping county-level mobility pattern changes in the United States in response to COVID-19. SIGSPATIAL Special.

[123] Dong E, Du H, Gardner L (2020). An interactive web-based dashboard to track COVID-19 in real time. Lancet Infect Dis.

[124] Lang TA, Altman DG (2015). Basic statistical reporting for articles published in biomedical journals: the 'statistical analyses and methods in the published literature' or the SAMPL guidelines. Int J Nurs Stud.

[125] Tong A, Sainsbury P, Craig J (2007). Consolidated criteria for reporting qualitative research (COREQ): a 32-item checklist for interviews and focus groups. Int J Qual Health Care.

[126] Moola S, Munn Z, Tufanaru C, Aromataris E, Sears K, Sfetcu R, Currie M, Qureshi R, Mattis P, Lisy K, Musy P-F (2017). Checklist for Analytical Cross Sectional Studies. The Joanna Briggs Institute.

[127] Howard Z, Somerville MM (2014). A comparative study of two design charrettes: implications for codesign and participatory action research. CoDesign.

[128] (2020). Social Determinants of Health. World Health Organization.

[129] McGinnis JM, Stuckhardt L, Saunders R, Smith M (2013). Best Care at Lower Cost: The Path to Continuously Learning Health Care in America.

[130] Etheredge LM (2007). A rapid-learning health system. Health Aff (Millwood).

[131] Greene SM, Reid RJ, Larson EB (2012). Implementing the learning health system: from concept to action. Ann Intern Med.

[132] Basole RC, Braunstein ML, Sun J (2015). Data and analytics challenges for a learning healthcare system. J Data Inform Qual.

[133] Berry I, Soucy JR, Tuite A, Fisman D, COVID-19 Canada Open Data Working Group (2020). Open access epidemiologic data and an interactive dashboard to monitor the COVID-19 outbreak in Canada. Can Med Assoc J.

[134] McGowan J, Sampson M, Salzwedel D, Cogo E, Foerster V, Lefebvre C (2016). Press peer review of electronic search strategies: 2015 guideline statement. J Clin Epidemiol.

[135] Keshavjee K, Mirza K, Martin K (2015). The next generation EMR. Stud Health Technol Inform.

[136] Mühlbacher T, Piringer H (2013). A partition-based framework for building and validating regression models. IEEE Trans Vis Comput Graph.

[137] Potter K, Wilson A (2009). Ensemble-Vis: A Framework for the Statistical Visualization of Ensemble Data. IEEE International Conference on Data Mining Workshops.

[138] Sedlmair M, Heinzl C, Bruckner S, Piringer H, Möller T (2014). Visual parameter space analysis: a conceptual framework. IEEE Trans Vis Comput Graph.

[139] Harrower M (2003). Tips for designing effective animated maps. CP.

[140] Shahar Y, Musen MA (1996). Knowledge-based temporal abstraction in clinical domains. Artif Intell Med.

[141] Müller E, Günnemann S, Assent I, Seidl T (2009). Evaluating clustering in subspace projections of high dimensional data. Proc VLDB Endow.

[142] Shneiderman B (1996). The Eyes Have It: a Task by Data Type Taxonomy for Information Visualizations. IEEE Symposium on Visual Languages.

[143] Wasi P (2000). Triangle That Moves the Mountain and Health Systems Reform Movement in Thailand.

[144] Bryant T (2010). Social Determinants of Health: The Canadian Facts.

[145] Tate AR (2011). Developing Quality Scores for Electronic Health Records for Clinical Research: a Study Using the General Practice Research Database. Proceedings of the First International Workshop on Managing Interoperability and Complexity in Health System.

[146] Volz J (2009). Silk-a link discovery framework for the web of data. Ldow.

[147] (2012). High Performance Visualization.

[148] Tableau.

[149] Qlik.

[150] Power BI. Microsoft.

