# Supplementary material for: Visual Analytic Tools and Techniques in Population Health and Health Services Research: Scoping Review
Source: J Med Internet Res. 2020 Dec 3;22(12):e17892. doi: 10.2196/17892 (PMC7716797; doi:10.2196/17892)
Supplement: Multimedia Appendix 2 [file jmir_v22i12e17892_app2.docx]

**Multimedia Appendix 2: Medical Literature Analysis and Retrieval System Online (MEDLINE) Search strategy**

| **#** | **Searches** | **Results** |
| --- | --- | --- |
| 1 | "visual* analytic*".tw,kf. | 498 |
| 2 | ((health or healthcare) adj (analytic* or analy*)).ti,kf. | 157 |
| 3 | data visualization/ | 5 |
| 4 | (visual* adj analy*).tw,kf. | 4448 |
| 5 | (information adj visual*).tw,kf. | 446 |
| 6 | (data adj visual*).tw,kf. | 1616 |
| 7 | (visual* adj platform?).tw,kf. | 118 |
| 8 | (visual* adj dashboard?).tw,kf. | 20 |
| 9 | (visual* adj representation?).tw,kf. | 1711 |
| 10 | (interactiv* adj3 (map* or graph*)).tw,kf. | 1041 |
| 11 | (visual* and (analy* or information* or data or platform? or dashboard? or representation?)).ti,kf. | 11707 |
| 12 | or/3-11 | 18488 |
| 13 | Big data/ | 125 |
| 14 | big data.tw,kf. | 4900 |
| 15 | ((health or healthcare) adj3 data).tw,kf. | 32400 |
| 16 | exp Health services research/ | 156248 |
| 17 | ((health or healthcare) adj3 (research* or data or service? or deliver*)).tw,kf. | 234548 |
| 18 | exp Health Services Accessibility/ | 103235 |
| 19 | Health Planning/ | 21374 |
| 20 | exp Regional Health Planning/ | 38865 |
| 21 | Health Resources/ | 11994 |
| 22 | Healthcare Disparities/ | 13936 |
| 23 | ((health or healthcare) adj3 (equit* or equalit* or inequit* or inequalit* or disparit*)).tw,kf. | 28701 |
| 24 | ((health or healthcare) adj3 (plan? or planning?)).tw,kf. | 28398 |
| 25 | ((health or healthcare) adj3 evaluat*).tw,kf. | 28202 |
| 26 | ((health or healthcare) adj3 (utiliz* or utilis* or access* or resourc*)).tw,kf. | 68797 |
| 27 | ((health or healthcare) adj3 monitor*).tw,kf. | 9584 |
| 28 | Population Health/ | 374 |
| 29 | exp Population Characteristics/ | 1823783 |
| 30 | (population* adj3 health*).tw,kf. | 40191 |
| 31 | (population* adj3 (characteristic? or demographic?)).tw,kf. | 29135 |
| 32 | exp Population Surveillance/ | 64189 |
| 33 | Public Health Systems Research/ | 18 |
| 34 | surveillance.tw,kf. | 159662 |
| 35 | biosurveillance.tw,kf. | 260 |
| 36 | exp Public Health/ | 7247194 |
| 37 | (public* adj3 health*).tw,kf. | 249554 |
| 38 | epidemiolog*.tw,kf. | 381559 |
| 39 | (administrative adj2 (data or database)).tw,kf. | 11164 |
| 40 | or/13-39 | 8019705 |
| 41 | 12 and 40 | 5689 |
| 42 | 1 or 2 or 41 | 6185 |
| 43 | limit 42 to yr="2005 -Current" | 4719 |
| 44 | limit 43 to English language | 4563 |
