# Supplementary material for: Visual Analytic Tools and Techniques in Population Health and Health Services Research: Scoping Review
Source: J Med Internet Res. 2020 Dec 3;22(12):e17892. doi: 10.2196/17892 (PMC7716797; doi:10.2196/17892)
Supplement: Multimedia Appendix 3 [file jmir_v22i12e17892_app3.pdf]

Author and year	Country	Problem analyzed	Settings			Target audience					
			Government; ministry; health unit	*Academic*	*Industry*	*Population/ public health practitioners*	*Clinicians*	*Data Scientists*	*Industry: software, pharmaceutical, insurance*	*Public and patient groups*	*Policy and decision makers*
Abusharekh et al, 2015 [67]	Canada	Analyze lab data to generate a physician scorecard outlining their order patterns.	x	x		x	x	x			
Afzal et al, 2011 [85]	USA	Development of a predictive VA suite for decision history and navigation to support simultaneous comparison of mortality and infection rates.	x	x		x		x			
Ali et al, 2016 [68]	Pakistan	Developing a service-oriented visual analytics architecture for ID surveillance, which provides automated mechanisms for ID data acquisition, outbreak detection and epidemic response management.		x		x					
Alonso et al, 2012 [92]	Brazil	Using EPIPOI to explore epidemological datasets for trends, seasonality, anomalies, comparisons across geographical locations.	x	x		x	x				
Antoniou et al, 2010 [93]	Greece	Visualizing epidemiologic data on autoimmune diseases for geographic and statistical parameters from publications.		x		x	x	x			
Antunes de Mendonca et al, 2015 [86]	Brazil	Present map to visualize information about the infestation of Aedes aegypti (dengue vector) in the municipality of Cuiabá, based on open data mashup.	x							x	
Baytas et al, 2016 [80]	USA	Developing a tool employing Sparse Principal Component Analysis for EHR cohorts to identify clinical features and visually present them in multiple	x			x	x	x			
Benis et al, 2017 [89]	Isreal	Analyzing ADHD data within Clalit Health Services for socio-demographic and ADHD filled prescription data	x	x		x	x				
Bryan et al, 2015 [64]	USA	Developing an epidemic simulation and predictive analytic system for non-experts	x	x		x	x	x			
Byrd et al, 2016 [94]	Canada	To test efficacy and reliability of the Twitter data to track influenza, and consider if a real time monitoring system can be made.		x		x		x			
Castronovo et al, 2009 [77]	USA	Developing dynamic mapping techniques for performing exploratory analysis of disease dynamics and for generating testable hypotheses.	x			x					

Author and year	Country	Problem analyzed	Settings			Target audience					
			Government; ministry; health unit	*Academic*	*Industry*	*Population/ public health practitioners*	*Clinicians*	*Data Scientists*	*Industry: software, pharmaceutical, insurance*	*Public and patient groups*	*Policy and decision makers*
Chen et al, 2016 [95]	Taiwan	Developing an online predictive analytic tool for identify hot spots of dengue fever.	x			x					
Chorianopoulos et al, 2016 [96]	Greece	To use user submitted data with high resolution geo-location to view real time symptom reports by area.		x		x	x	x			
Dagliati et al, 2018 [66]	Italy	Developing a dashboard-based system for type 2 diabetes clinical decision making and predictive analytics for complications among populations.	x			x	x	x			
Deodhar et al, 2015 [65]	West Africa	To design epidemic forecasting tools for global diseases, for decision makers through a web-based system.	x			x		x			
Garcia-Marti et al, 2017 [97]	Netherlands	Studying spatio-temporal distribution of tich bites using frequent pattern mining to decrease the incidence of Lyme disease.		x		x		x			
Gligorijevi et al, 2017 [98]	North Macedonia	Developing a prototype for mapping patient travel for a certain condition to visualize access.	x			x		x			
Gotz et al, 2014 [76]	USA	To present a novel VA technique for visual exploration of patterns mined from retrospective clinical patient data.		x	x	x	x	x	x		
Guo et al, 2007 [69]	USA	Propose a visual analytical approach to explore spatial interaction patterns in very large datasets of individual-based population movements and simulated pandemic spreads.		x		x		x			
Haque et al, 2014 [99]	Canada	Developing a Business Intelligence (BI) tools based system for monitoring health conditions and services in a rural health authority	X			x	x	x			
Hardisty et al, 2010 [100]	USA	Developing a tool for better understanding spatio-temporal correlations in data.	x	x		x		x			
Huang et al, 2015 [101]	Taiwan	Developed a visual mining system to support exploratory data analysis of multi-dimensional categorical EMR	x			x	x	x			
Hund et al, 2016 [90]	Germany	Presenting Sub-VIS, an interactive tool to demonstrate the potential of subspace analysis for the interpretation of highdimensional health data.		x		x	x	x			
Ji et al, 2012 [102]	USA	Developing a tool for visualizing epidemic spread spatio-temporally using Twitter data.		X		x		x			

**Author and year**	**Country**	**Problem analyzed**	**Settings**			**Target audience**					
			Government; ministry; health unit	***Academic***	***Industry***	***Population/ public health practitioners***	***Clinicians***	***Data Scientists***	***Industry: software, pharmaceutical, insurance***	***Public and patient groups***	***Policy and decision makers***
Ji et al, 2013 [81]	USA	Developing novel Twitter sentiment classification tool to gauge the users' degree of concern (DOC) for diseases.		x		x		x		x	x
Jiang et al, 2016 [103]	USA	Develop a web-based healthcare data visualization system, based on a Notifiable Condition Detector (NCD) use case, with 2 new visualizations.	x	x		x		x			
Jinpon et al, 2017 [83]	Thailand	Design and useability testing of a web-based tool for patterns of community well-being in Thailand.	x			x		x			
Kaieski et al, 2016 [104]	USA	Presents tool for studying co-variance between variables, while visualizing results.	x	x		x		x		x	
Katsis et al, 2017 [105]	USA	Present big data techniques for exploring public health datasets for health outcomes.		x		x		x			
Kostkova et al, 2014 [75]	UK	Proposed a new epidemic monitoring system to better automate early warning, cross validation of signals for outbreak detection and visualization of results on an interactive dashboard.		x		x		x			
Kruzikas et al, 2014 [106]	USA	Developing an agent based model to estimate the impact of resource allocation decisions on population health and health care costs.	x			x		x			x
Lavrac et al, 2007 [70]	Slovenia	Propose an innovative use of data mining and visualization techniques for decision support in planning and regional level management of Slovenian public health-care.	x	x		x		x			
Lu et al, 2017 [71]	UK	Modeling cancer survival and developing a framework for visual analytics.	x	x		x	x	x			
Luo et al, 2016 [78]	France	Proposes a new geo-social interaction pattern framework for disease control and building a VA system for simulating various scenarios.		x		x		x			
Maciejewski et al, 2010 [107]	USA	Introduce a VA approach for exploring cancer care statistics to reduce the small area and number problem.		x				x			
Maciejewski et al, 2011 [79]	USA	Develop VA toolkit for analyzing the effect of decision measures implemented during a simulated pandemic influenza scenario.	x	x		x		x		x	x

Author and year	Country	Problem analyzed	Settings			Target audience					
			Government ; ministry; health unit	*Academic*	*Industry*	*Population/ public health practitioner s*	*Clinicians*	*Data Scientists*	*Industry: software, pharmaceut ical, insurance*	*Public and patient groups*	*Policy and decision makers*
Marek et al, 2015 [108]	Czech Republic	Use of Google Earth as geovisual analytic tool to understand spatio-temporal patterns for campylobacteriosis in Czech Republic		x		x		x			
Mitrpanont et al, 2017	Thailand	To implement large displays for complex visualizations from	x			x	x	x			x
Mittelstadt et al, 2014 [110]	Italy	Devloped a VA system with scalable interfaces to analyze low frequency adverse drug reactions	x			x	x	x			
Ozkaynak et al, 2015 [111]	USA	To analyze and compare workflows in emergency departments and satellite centers using vis and Markov Chains to reveal sequence patterns.	x	x		x	x	x			
Park et al, 2018 [112]	USA	Compare discussion topics in publicly accessible online mental health communities for three conditions using text mining.		x		x		x			
Perer et al, 2015 [113]	USA	Derive patterns of diagnoses and treatments of hyperlipidemic patients cohort with hypertension and diabetes pre-conditions from EMR data.		x	x	x	x	x	x		
Proulx et al, 2006 [114]	USA	Demonstrtae analytic synergy achieved by comining GeoTime's ge-temporal analytic capability for triage and sense making.	x	x		x		x			
Shaban-Nejad et al, 2017 [84]	Canada	Develop a knowledge based platform using population health indicators to support decision making.	x	x		x					
Soulakis et al, 2015 [115]	USA	Developing a graph-based analysis method to identify healthcare interactions for hospitalized patients with heart failure.	x			x	x				
Tate et al, 2014 [87]	UK	Develop tool for users to identify patients for recruitment into randomized controlled trials (RCT) from primary care administrative		x		x		x			
Tilahun et al, 2014 [88]	Germany	Evaluate Linked Data technologies as potential options for health information visualization, and retrieval systems development.	x			x		x			x
Toddenroth et al, 2014 [116]	Germany	Propose strategies to adapt heat maps for associations and causal effects within EMR data.	x			x	x	x			

**Author and year**	**Country**	**Problem analyzed**	**Settings**			**Target audience**					
			Government; ministry; health unit	***Academic***	***Industry***	***Population/ public health practitioners***	***Clinicians***	***Data Scientists***	***Industry: software, pharmaceutical, insurance***	***Public and patient groups***	***Policy and decision makers***
Torres et al, 2012 [117]	USA	Present a VA system to help researchers explore patterns and form hypotheses from the NHANES dataset.	x			x		x			x
Widanagamaachchi et al, 2017 [72]	USA	Using optimized data structures and progressive visualization techniques, allowing users to interactively explore patient progression, using EHR data.		x		x	x	x			
Xing et al, 2010 [91]	Canada	Direct disease interactive pattern mining method to explore the NHANES data for finding interactions between diseases in the population.		x		x		x			
Xu et al, 2013 [73]	USA	Correlation mining for investigating variable subsets by use of heatmaps.	x	x		x	x	x			
Yan et al, 2013 [118]	China	Pilot implementation of an electronic surveillance system (ISS) for early detection of epidemics in rural China.	x	x		x		x			
Yu et al, 2017 [82]	USA	Present a visualization framework to aid decision makers to identify and optimize geographic variations in	x	x		x		x		x	x
Yu et al, 2018 [74]	China	Collecting and analyzing several datasets on PM2.5 air pollution, environments and public health in Beijing in order to discover the intrisic relations between factors.	x	x	x	x		x	x		
x = applicable category											
