# Supplementary material for: Visual Analytic Tools and Techniques in Population Health and Health Services Research: Scoping Review
Source: J Med Internet Res. 2020 Dec 3;22(12):e17892. doi: 10.2196/17892 (PMC7716797; doi:10.2196/17892)
Supplement: Multimedia Appendix 4 [file jmir_v22i12e17892_app4.pdf]

#	Author and year	Tool name; *and* base application	**Analytic capability**			**Goal of the application/method**	
			Descriptive analytics	***Predictive analytics***	***Visual exploration of complex datasets***	***Knowledge discovery/exploratory analysis***	***Decision support***
1	Abusharekh et al, 2015 [67]	H-Drive; information analytics based on R.	x	x		x	x
2	Afzal et al, 2011 [85]	Not mentioned.		x			x
3	Ali et al, 2016 [68]	ID-Viewer	x	x			x
4	Alonso et al, 2012 [92]	EPIPOI based on Matlab	x		x	x	x
5	Antoniou et al, 2010 [93]	dAUTObase	x			x	
6	Antunes de Mendonca et al, 2015 [86]	Based on Triplify, SQL, PHP, SPARQL EndPOint	x			x	
7	Baytas et al, 2016 [80]	PhenoTree	x		x	x	x
8	Benis et al, 2017 [89]	DisEpi, R based	x		x	x	x
9	Bryan et al, 2015 [64]	EpiSimS	x	x	x	x	x
10	Byrd et al, 2016 [94]	Not mentioned.	x		x		
11	Castronovo et al, 2009 [77]	Not mentioned.					
12	Chen et al, 2016 [95]	SaTScan software					
13	Chorianopoulos et al, 2016 [96]	Flutrack.org	x		x	x	x
14	Dagliati et al, 2018 [66]	MOSAIC dashboard; Data mining using R and Matlab; JavaScript; HTML; Google Charts for GUI.	x	x	x	x	x
15	Deodhar et al, 2015 [65]	EpiCaster	x	x	x	x	x
16	Garcia-Marti et al, 2017 [97]	Not mentioned.	x		x	x	x
17	Gligorijevi et al, 2017 [98]	Not mentioned.	x		x	x	x
18	Gotz et al, 2014 [76]	Not mentioned.	x		x	x	
19	Guo et al, 2007 [69]	Not mentioned.	x	x			x
20	Haque et al, 2014 [99]	Microsoft SQL Server's BI tool stack and ASP.NET	x		x	x	x
21	Hardisty et al, 2010 [100]	LISTA-Viz	x		x	x	
22	Huang et al, 2015 [101]	Not mentioned.	x		x	x	
23	Hund et al, 2016 [90]	Sub-VIS; based on d3.js2	x		x	x	
24	Ji et al, 2012 [102]	ESMOS (Epidemic Sentiment Monitoring System)	x		x	x	x
25	Ji et al, 2013 [81]	ESMOS (Epidemic Sentiment Monitoring System)	x				
26	Jiang et al, 2016 [103]	Health-Terrain	x		x	x	x
27	Jinpon et al, 2017 [83]	Community Well-Being Assessment System (CWBAS)	x		x		X
28	Kaieski et al, 2016 [104]	Vis-Health	x		x	x	
29	Katsis et al, 2017 [105]	Not mentioned.	x		x	x	
30	Kostkova et al, 2014 [75]	medi+board	x			x	x
31	Kruzikas et al, 2014 [106]	Not mentioned.		x			x
32	Lavrac et al, 2007 [70]	MediMap	x	x		x	x
33	Lu et al, 2017 [71]	Southampton Breast Cancer Data System (SBCDS)	x	x		x	x
34	Luo et al, 2016 [78]	GS-EpiViz	x			x	x
35	Maciejewski et al, 2010 [107]	Not mentioned.	x			x	x
36	Maciejewski et al, 2011 [79]	PanViz				x	x

#	Author and year	Tool name; *and* base application	**Analytic capability**			**Goal of the application/method**	
			Descriptive analytics	***Predictive analytics***	***Visual exploration of complex datasets***	***Knowledge discovery/exploratory analysis***	***Decision support***
37	Marek et al, 2015 [108]	R with spacetime, gstat and plotKML; and Google Earth	x			x	x
38	Mitrpanont et al, 2017 [109]	SAGE2	x		x	x	x
39	Mittelstadt et al, 2014 [110]	Not mentioned.	x			x	
40	Ozkaynak et al, 2015 [111]	EventFlow and Discrete Time Markov Chains	x			x	x
41	Park et al, 2018 [112]	Not mentioned.	x			x	
42	Perer et al, 2015 [113]	Care Pathway Explorer	x			x	x
43	Proulx et al, 2006 [114]	nSpace and GeoTime	x			x	x
44	Shaban-Nejad et al, 2017 [84]	Population Health Record (PopHR)	x		x		x
45	Soulakis et al, 2015 [115]	Not mentioned.	x			X	
46	Tate et al, 2014 [87]	TrialViz	x		x	x	x
47	Tilahun et al, 2014 [88]	Not mentioned.	x			x	x
48	Toddenroth et al, 2014 [116]	Not mentioned.	x			x	
49	Torres et al, 2012 [117]	Not mentioned.	x			x	
50	Widanagamaachchi et al, 2017 [72]	Not mentioned.	x	x		x	x
51	Xing et al, 2010 [91]	Not mentioned.	x			x	
52	Xu et al, 2013 [73]	Not mentioned.	x	x		x	x
53	Yan et al, 2013 [118]	ISS (syndromic surveillance system)	x			x	x
54	Yu et al, 2017 [82]	Patient-Provider Geographic Map	x				
55	Yu et al, 2018 [74]	Watson analytics	x	x		x	

x = applicable category
