# Supplementary material for: Visual Analytic Tools and Techniques in Population Health and Health Services Research: Scoping Review
Source: J Med Internet Res. 2020 Dec 3;22(12):e17892. doi: 10.2196/17892 (PMC7716797; doi:10.2196/17892)
Supplement: Multimedia Appendix 5 [file jmir_v22i12e17892_app5.pdf]

Author and year	DATA TYPE AND SOURCE												ANALYTIC METHODS		VISUALIZATION METHODS			
	**Data used and user case/s**	**Data sources**	**Structured**	**Unstructured**	**Semi-structured**	***Registry***	***Administrative/ survey***	***EMR/ EHR***	***ST***	***Sensor***	***Simulation***	***Web/Social media***	**Analytic ENGINE/ tools**	**Analytic methods/ algorithms**	**Visual Engine/ tool**	**Visual PRESENTATION**	***Specific features***	***Interactivity features***
Abusharekh et al, 2015 [67]	Requisitions for 15 most popular tests ordered in a 1-year period; over 15 million tests for around 200,000 patients ordered by over 2100 primary care physicians.	Single dataset/database.			x			x					Suite of analytic offering including Exploratory Analytics using data mining; Semantic Analytics to infer contextualized relationships ; Predictive Analytic modeling for future trends/outcomes; Information Analytics, using statistical methods.	K-means clustering; statistical methods for physician scorecard	Block modeling; heat maps; using K-means clustering in Apache Mahout.	Geo-spatial maps, dashboards, scorecards, 2D and 3D models and animations	Live lab utilization overview with Google Maps; Prediction based on order frequency. Order profile generation: showing clusters of tests ordered together, using Apache Mahout; displayed using test co-occurence matrix; Physician profile Generation: showing test order behavior.	Scorecards, maps and user input.
Afzal et al, 2011 [85]	Applied to 2 user cases of Rift Valley Fever and Influenza; simulation data used.	Simulation data.	x						x				Generates a large scale spatial simulations. Population and demographic data is provided as input. Simulation then outputs information on the number of sick and dead within a given population by areal unit and provides color coded geographical representations of the data.	Not mentioned..	Not given	Spatiotemporal model view: History tree view. Each symbol in the decision history tree represents the insertion point of the decision path. A unique color is assigned to each symbol and the corresponding decision path.	As users interact in the model view, the decisions made generate a history tree. Paths of the tree are plotted over time on the x-axis, with the y-axis representing the cumulative deviation from the baseline simulation.	Mousing over on a node brings up a thumbnail view of the decision measures implemented at that point in the simulation. Legend symbols represent mitigative response measure types.
Ali et al, 2016 [68]	Muti-source data including from teriary hospitals, drug sales and other sources.	Multiple datasets.									x		Dynamic syndromic classification module is developed using fuzzy logic and artificial neural networks; and follows hierarchal neuro-fuzzy approach for classification. Epidemic outbreak detection uses SaTScan that allows detection of geographical disease clusters; The Disease forecast module input features include, auto-regressive terms acquired using partial auto-correlation function and using Seasonal-Trend Decomposition using Loess (STL).	Not mentioned..	Geo-spatial analysis routines consist of spatial queries, e.g. k-nearest neighbour, range queries, reverse nearest neighbour etc., for efficient resource allocation, health care load management, and identification of potential geographical sites seeking health care facilities.	Choropleth map shows the variation in disease spread along geographic boundaries. Heat-map provides a comprehensive picture of disease severity by showing variation in ID concentration without any distinction of geographical boundaries.	Supports real-time ID data visualization alongside syndrome specific information gathered at the instance of patient registration.	Pan, tilt, zoom, region selection functions in the maps

Author and year	DATA TYPE AND SOURCE												ANALYTIC METHODS		VISUALIZATION METHODS			
	**Data used and user case/s**	**Data sources**	**Structured**	**Unstructured**	**Semi-structured**	***Registry***	***Administrative/ survey***	***EMR/ EHR***	***ST***	***Sensor***	***Simulation***	***Web/Social media***	**Analytic ENGINE/ tools**	**Analytic methods/ algorithms**	**Visual Engine/ tool**	**Visual PRESENTATION**	***Specific features***	***Interactivity features***
Alonso et al, 2012 [92]	Pneumonia and influenza datasets from Department of Vital Statistics from the Brazilian Ministry of Health; dataset details not provided.	Single dataset/database.	x						x				Matlab	Not mentioned..	Matlab based package	Insights on epidemiologic trends and patterns over time.	Time series, seasonality for epidemics, anomalies in data, scatterplots	User input for time period and other parameters.
Antoniou et al, 2010 [93]	Data mined more than 100 publications	Publications		x									Database queried using PivotViewer based on MS Silverlight Technology.	Not mentioned..	Silverlight based PivotViewer Application	Cards presenting populations and epidemilogical results, with data on the side; geographic distributors curated by the databases.	Menus and clickable options to form queries.	Checkboxes and menus.
Antunes de Mendonca et al, 2015 [86]	Open government health and other databases from Mato Grosso Health State Department and Cuiaba Health Department	Multiple databases	x				x		x				Triplify, SQL, PHP, SPARQL EndPOint	Protégé used for ontology creation, Triplify used for mapping of relational data by means of SQL	Google map using API Exhibit	Browser based display of mapping of instances of Dengue Fever across city of Cuiaba using Google map	API can be reused to collect other government data and display another topic of interest	Details on mouse hover
Baytas et al, 2016 [80]	Public and private EHRs; two data sets of 101,767 and 223,076 patients in the US.	Multiple databases.	x	x	x			x					Interactive heirarchical: 1. SPCA is applied to non-zero loading values, forming clinical input features. 2. Features in first category are refined for sub-populations (expanded to a tree structure).	SPCA based algorithms.	Web based application	Cohorts and sub-cohorts visualized in a circular tree, with nodes and sub-nodes	Each node gives a structured phenotype and a stable subcohort characterized by this phenotype.	User input for various cohort selection through multiple filters
Benis et al, 2017 [89]	EHR of the Clalit Health Services, Israel with over 4.4 million members.	Single dataset/database.	x				x						R with doParallel and gplots	Hierarchical clustering	R Package gplots	Heatmap		Heatmap generated is not interactive; user input is interactive
Bryan et al, 2015 [64]	Simulating chiknngunya on the Washington D.C. metro area, with about 500,000 people.	Simulation data	x				x				x		R scripts	Predictive models using statistical methods for goodness of fit.	EpiSimS	Timeline, scatter plot, bar chart, radar plot, heatmaps		Not clear.
Byrd et al, 2016 [94]	2 million tweets over 1 month using the Twitter Streaming API with location filters for influenza	Twitter data		x								x	Stanford CoreNLP	Naïve Bayes, Maximum Entropy, and a Dynamic Language Model classifier	Open- Layers 3 (JavaScript)	Map and pie chart		Interactive map with ability to select tweets on their location.

Author and year	DATA TYPE AND SOURCE												ANALYTIC METHODS		VISUALIZATION METHODS			
	Data used and user case/s	Data sources	Structured	Unstructured	Semi-structured	*Registry*	*Administrative/ survey*	*EMR/ EHR*	*ST*	*Sensor*	*Simulation*	*Web/Social media*	Analytic ENGINE/ tools	Analytic methods/ algorithms	Visual Engine/ tool	Visual PRESENTATION	*Specific features*	*Interactivity features*
Castro novo et al, 2009 [77]	Hospitalization records for Salmonella infections abstracted from Centers for Medicare and Medicaid Services for all Medicare recipients aged 65 or above in the contiguous U.S. for 2002 (Alaska, Hawaii, Virgin Islands and Puerto Rico were excluded from the analysis); weather data from the PRISM group at Oregon State University.	Multiple datasets.	x				x	x					ESRI ArcMap	Data abstraction, aggregation and mapping done using different datasets	ESRI ArcMap	Dynamic maps	Can track multiple locations of disease occurrence simultaneously, over time.	Interface consisting of control buttons for stop, play, move forward or back one frame, and replay.
Chen et al, 2016 [95]	57,516 confirmed DF cases from the Taiwan Open Data Platform; and demographic information from the Government	2 datasets	x						x				Statistical modeling; sensitivity and specificity analysis;	Poisson modeling with prospective space-time statistic computed weekly; Monte Carlo hypothesis testing.	PHP (version 5.5), JavaScript (OpenLayers and Highcharts libraries), and HTML	Interactive map		Interactive interface for users to manipulate relevant parameters of scan statistics and visualize the weekly relative risk of DF at the village level.
Choria nopoul os et al, 2016 [96]	Twitter API data, including tweets and geolocation at time of tweet	Twitter data		x								x	Flutrack API	Linguistic filtering	Google map overlay	Real time updated Google Map overlay with of Flu-like symptom tweets	Real time linked data	Selectable locations, zoom levels, and anonymous tweets
Dagliat i et al, 2018 [66]	ICSM EHR and the local public health agency, Agenzia Tutela Salute (ATS). 10 years retrospectively, 6 months prospectively.	2 datasets	x				x	x					Temporal abstractions, careflow mining, drug exposure pattern, risk prediction models of Type 2 Diabetes related complications.	3 modules: data; logical for querying/mining; graphical. Various algorithms, which were part of the JTSA, CFM and other tools.	Google Charts	3 main sections: metabolic control: assessment of HbA1c, BP, diet, BMI, risk calculations of cardio- and micro-vascular; frequent temporal patterns of diet and weight information; drug purchase patterns for classes.	Visualization of long term complication episodes, drug purchases, patients grouped by demographic and clinical variables	Filters for age and BMI

**Author and year**	**DATA TYPE AND SOURCE**												**ANALYTIC METHODS**		**VISUALIZATION METHODS**			
	**Data used and user case/s**	**Data sources**	**Structured**	**Unstructured**	**Semi-structured**	***Registry***	***Administrative/ survey***	***EMR/ EHR***	***ST***	***Sensor***	***Simulation***	***Web/Social media***	**Analytic ENGINE/ tools**	**Analytic methods/ algorithms**	**Visual Engine/ tool**	**Visual PRESENTATION**	***Specific features***	***Interactivity features***
Deodhar et al, 2015 [65]	Multiple (Ebola data from WHO, CDC, etc); Sierra Leone data.	Multiple sources	x				x	x	x			x	Different pipelines for pulling in data from different sources, forecasting, and visualisation.	Modeling silmuations; epicaster module suports aggregate based models, using ordinary differential equations and agent based models.	EpiCaster's Web-enabled User Interface	Users can view the current and forecasted state of various epidemics through high-resolution maps and plots at different spatio-temporal levels.	Interactive timeline: Range of weeks (last four to two weeks in future); Interactive heat map: GIS enabled infection levels aggregated at multiple geographical levels. Plots (Epicurves): infection trends in a region over a time period, as well as the peak infection count in the specified time period	Interactive map
Garcia-Marti et al, 2017 [97]	Volunteer reported tick bite data between 2006-14 (NK, 9256; TR, 24,584); environment data (weather, vegetation, land)	Multiple sources: environmental data, weather data, vegetation indoctors, land use and soil data	x						x				Frequent pattern mining using SPMF, Java based open source platform.	AprioriClose for pattern recognition; Jenks Natural Breaks (JNB) algorithm for classifying non-categorical data. Unsupervised method.	Python for heat maps, Javascript for ring maps.	Three types of graphical elements: (1) heat maps to summarize the patterns; (2) interactive ring maps for general overview of the relevance of features in patterns; and (3) maps to display two selected patterns in geographic space.		Interactive ring maps
Gligorijevi et al, 2017 [98]	EHR data from Macedonian government records of about 2,700 clinics, and 7600 doctors; tested on 2 datasets 29854 patients in autoimmune; 124032 in cancer datasets.	Single dataset/database.	x					x					Data compiler based on .NET compiler platform.	Not used.	OpenStreetMap for maps, D3.js and Chart.js for visualization	Interactive map with filters showing patients, migrations and distance, comparable between municipalities.	Filters data based on ICD 10 codes; provides map with statistics	Filters and drill downs; distances covered.
Gotz et al, 2014 [76]	3 Cohorts: hypothyroid, heart failure and hypertensive patients; details not provided.	Single EMR database.		x	x			x					Temporal data mining from time point based event sequences	Sequential pattern mining	Visual querying methods specifying milestones, preconditions and an outcome measure.	Milestone timeline, pattern diagrams with scatter plots and trend line, interactive user selection for variables.	Size and color of circles indicate the severity of outcome and patterns mined. Circle color indicates odds ratios, side bar shows full set of statistics including p-values for the patterns. Support for temporal comparison of outcomes a key feature.	Mouse hover/click shows details.

Author and year	DATA TYPE AND SOURCE												ANALYTIC METHODS		VISUALIZATION METHODS			
	**Data used and user case/s**	**Data sources**	**Structured**	**Unstructured**	**Semi-structured**	***Registry***	***Administrative/ survey***	***EMR/ EHR***	***ST***	***Sensor***	***Simulation***	***Web/Social media***	**Analytic ENGINE/ tools**	**Analytic methods/ algorithms**	**Visual Engine/ tool**	**Visual PRESENTATION**	***Specific features***	***Interactivity features***
Guo et al, 2007 [69]	Simulated data: Human activities 1.6m with181,267 locations in Portland metropolitan area for a normal day and a simulated pandemic outbreak for a 100-day period.	2 simulated data sources	x						x		x		Graph partitioning; matrix visualization and flow map, interactively linked to the reorderable matrix, for patterns; tools not mentioned.	Graph parititioning uses the Guibas–Stolfi algorithm	Not mentioned.	Modified flow maps	Interactive user selection for matrices and details.	Ability to select parts of matrix/graph to visualize maps/spread.
Haque et al, 2014 [99]	BC Northern Health's database of about 300,000 people.	Multiple datasets	x				x		x				MS SQL Server's BI tool stack, ASP.net, OLAP cube, SQL reporting service	Not mentioned.	MS SQL Reporting Services	Dashboard with Population profile; patient profile; Case Mix profile	Population profile: population pyramid, minorities (horizontal bar graph), income distribution, (pie), dependency rates (pie), graduation (pie). Patient profile: Misc. stats (table), beds in specialities (horizontal bar), annual potential years of life lost (pie), chonic diseases (pie). Case Mix Group profile: Table comparing Northern and BC ranking of health conditions.	Not properly explained.
Hardisty et al, 2010 [100]	2 datasets: 51 US states and 3105 counties, using Google Flu trens data for H1N1 pandemic 2009.	Multiple datasets	x						x				Java libraries in the GeoViz Toolkit	LISTA VIZ algorithm - details not provided.	e LISTA-Viz component allows interactive exploration of data and statistical significance of ST patterns.	Histograms with corresponding chloropleth maps; statistical significance showed through trend lines and R2 statistics.		Buttons for different stats.
Huang et al, 2015 [101]	Taiwan's National Health Insurance Research Database (NHIRD), a longitudinal database with ICD-9-CM codes for disease identification as well as procedures. Extracted 14,567 CKD patients who had eleven common comorbidities.	Single dataset/database.		x	x			x					Python based; with Java and HTML 5 for the web interface.	Uses a combination of frequency based cohort clustering; hierarchical clustering for events; and variance based association filtering.	Cohort based trajectories network model.	Sankey style diagrams	2 views: Trajectory Summary view shows patient characteristics.	Summary shows more information on patient metadata;

Author and year	DATA TYPE AND SOURCE												ANALYTIC METHODS		VISUALIZATION METHODS			
	**Data used and user case/s**	**Data sources**	**Structured**	**Unstructured**	**Semi-structured**	***Registry***	***Administrative/ survey***	***EMR/ EHR***	***ST***	***Sensor***	***Simulation***	***Web/Social media***	**Analytic ENGINE/ tools**	**Analytic methods/ algorithms**	**Visual Engine/ tool**	**Visual PRESENTATION**	***Specific features***	***Interactivity features***
Hund et al, 2016 [90]	Vaccination dataset from a family practice in Osijek, Croatia during 2003/2004; 35 male and 58 female persons aged between 50 and 89 years, with 61 dimensions describing clinical parameters	Single dataset/database.	x					x					Uses subspace clustering methods with experiments using decision trees, bayes classification and random forest.	Analyzes every subspace cluster independent of its association to a specific clustering structure or algorithm; uses the detected subspaces of the OpenSubspace Framework	3 level exploratory interactive tool.	First level gives an overview of clustering by bar charts and heat map; Second level allows selection of clusters resulting in aggregation tables; Third level allows looking at each record using table lens view.		Mouse hover gives details in different views.
Ji et al, 2012 [102]	2605 tweets in 2011 for listeria outbreak	Twitter data		x								x	ETL system: Data collector based on Twitter search and streaming APIs and phirehose libraries provided by 140dev. Relational database created using PHP.	Not mentioned.	Google Map API	Static map, Filter map with granularity, influence and timeline filters.	Maps providing different levels of granularity according to spatio-temporal variables. Colored circles provide tweet frequency according to state, country and world levels.	None mentioned.
Ji et al, 2013 [81]	645 Tweets from 2011-09-26 to 2011-09-28	Twitter data		x								x	ML Classification system, with 4 point Likert scale for sentiments	Tested multiple including Naïve Bayes, Multinomial Naïve Bayes, and Support Vector Machines	Not mentioned.	Timeline charts; concern maps (simple maps with color coding showing tweets patterns)	Concern maps showing geography of the country and level of concern.	Dropdown, timescale selection.
Jiang et al, 2016 [103]	Regenstrief Institute database 833,710 public health notifiable cases, 439,547 unique patients across ten years. For text mining, 325,791 clinical notes with discharge summaries, labs, histories.	Single dataset/database.				x		x	x				Data mining for association maps; text mining for term correlations.	NLP for named entity recognition (NER); stemming and concept clustering algorithms; Term correlations using tf-idf vector space model; association mining algorithm for association graphs.	Health Terrain (based on Java)	Split screen dashboard visualization that incorporates "Spatial Texture-Based" visuals and "Spiral Theme Plots"	STB visuals allow texturing of shaded areas and offset counter lines to denote attribute changes over time. Spiral theme plots allow plotting patients and diseases over time while setting a significant attribute (e.g age) as the radius	User inputs, selecting diseases, time ranges, additional info, splitting screen, choosing visualization methods etc.

**Author and year**	**DATA TYPE AND SOURCE**												**ANALYTIC METHODS**		**VISUALIZATION METHODS**			
	**Data used and user case/s**	**Data sources**	**Structured**	**Unstructured**	**Semi-structured**	***Registry***	***Administrative/ survey***	***EMR/ EHR***	***ST***	***Sensor***	***Simulation***	***Web/Social media***	**Analytic ENGINE/ tools**	**Analytic methods/ algorithms**	**Visual Engine/ tool**	**Visual PRESENTATION**	***Specific features***	***Interactivity features***
Jinpon et al, 2017 [83]	22,046 participants from 6,592 families.	Pooled data from multiple sites in a province.	x				x						MySQL, PHP, Highcharts JS, and Google Maps. Apache web server 2.2.8 and MySQL 5.0.51b used as web server and database management system.	Highcharts JS for interactive calculations and visualizations.	Highcharts JS adds interactive calculations. Google Maps is used to visualize interesting factors and to plan routes.	Pie, stacked bar, and population pyramid charts. Community well-being scores in bar chart, spider chart, table, and text summaries	Demographic reports are shown in pie, stacked bar, and population pyramid charts. Community well-being scores from all nine sub-districts visualized in drill-down bar chart, spider chart, table, and text summary formats	Calculations in Highcharts JS; Google maps API; drill down ability on most charts for well being scores.
Kaieski et al, 2016 [104]	Public health data obtained from Brazilian Sinan and BDMEP (Meteorological Database for Education and Research), from 2003-2012, 10 years, 7 state capitals, 5040 total records.	Single dataset/database.	x						x				Principal Component Analysis (PCA), resulting in a linear orthogonal regression.	Algorithm based on PCA	Open source tools; specific tool not mentioned.	Heat maps using geo-referenced time series; pie chart overlay on maps.	Heat maps show concentration of events; pie chart showing dependency relationship.	Filters, checkboxes and drop downs
Katsis et al, 2017 [105]	San Diego County data: First dataset contained 3,818 health outcome indicators; second dataset with 22,712 census tract indicators.	2 datasets	x				x		x				Random forest and extra-trees classifier combibintation through a bagging based approach; predictive modelling using ML; partitioned clustering method	Apriori algorithm; k-medioids clustering	Not mentioned.	Regional profile with maps and demographic breakdowns using pie charts; small mutiples vis for hospitalization rates; correlation matrix	Health oucome indicator breakdown for each region; and sub-regions. Heat map within the small multiples vis.	Not mentioned.
Kostkova et al, 2014 [75]	Simulation using 3 datasets from the swine flu 2009 pandemic (HPA surveillance, Google news, Twitter)	Multiple datasets and sources	x	x								x	html5/JavaScript web application	Monitoring and detection of multiple channels using different computational methods modularly provided (data mining, NLP, ML, data science, complex systems, social networks, etc). Newly identified signals in each data stream are validated during which their reliability coefficient is adjusted.	html 5/Javascript based web app	Dashboard visualization with pie, graph and maps.	Not provided.	Timeline on dashboard with filters

Author and year	DATA TYPE AND SOURCE												ANALYTIC METHODS		VISUALIZATION METHODS			
	Data used and user case/s	Data sources	Structured	Unstructured	Semi-structured	*Registry*	*Administrative/ survey*	*EMR/ EHR*	*ST*	*Sensor*	*Simulation*	*Web/Social media*	Analytic ENGINE/ tools	Analytic methods/ algorithms	Visual Engine/ tool	Visual PRESENTATION	*Specific features*	*Interactivity features*
Kruzikas et al, 2014 [106]	Simulation data using facilities and socio-demographic characteristics	Simulation data					x				x		Agent based modeling; Calculation engine comprises of algorithms from the GE Healthcare's CAD Diagnostic Optimization Model for Patients with Stable Chest Pain developed for the NIH	Utilizing demographic, epidemiologic, and economic data, the model then simulates associated health and economic effects, including QALYs, mortality, diagnostic and treatment costs, ROI and annual provider profit.	Mad*Pow using HTML5/CSS, Javascript, and Ruby	The ABMS model then integrates with a data visualization application to display inputs and outputs and to enable hospital configuration scenario evaluations through graphical representation. In the data visualization, users select parameters of interest for heat mapping and subsequently can evaluate the impact of new hospital configuration scenarios	The tool enables multiple scenarios to be computed and compared to inform decision-making	Click-through screens where clinical or health outcomes are displayed concurrently
Lavrac et al, 2007 [70]	Datasets from of 11 community health centers of the Celje region, including health care providers, out patient health care statistics, and medical status databases.	Multiple datasets	x				x						Clustering and classification	Decision tree learning algorithm-J48 WEKA implementation of the C4.5 learner	Not mentioned.	Different access maps for services	Enables visualization of areas of slovenia with low CHC access capacity.	Not mentioned.
Lu et al, 2017 [71]	Breast Cancer data from SBCDS, more than 17,000 patients since 1970s.	Multiple databases	x					x					Weka; for sequential pattern mining and classification.	Sequential Patterns Graph; Weka Decision Tree J48	Tableau and Weka.	Decision trees, line graphs, bar graphs, pie charts using Tableau; Patient Timeline visualization	Survival analysis comparisons with treatment, groups, and other other variables.	Roll up, drill downs, others
Luo et al, 2016 [78]	77,602 contact events; Face-to-face interactions among 242 individuals including 232 children and ten teachers, across ten classes over 2 days in a French primary school.	Single dataset/database.	x								x		Java universal network/graph framework (JUNG)	Agent based modelling using matrix; details not provided.	JFreeChart	GS-EpiViz consists of four major components: display panel, control panel, xy plot, and matrix view.	Allows the identification of human interaction patterns	Panels.

Author and year	DATA TYPE AND SOURCE												ANALYTIC METHODS		VISUALIZATION METHODS			
	**Data used and user case/s**	**Data sources**	**Structured**	**Unstructured**	**Semi-structured**	***Registry***	***Administrative/ survey***	***EMR/ EHR***	***ST***	***Sensor***	***Simulation***	***Web/Social media***	**Analytic ENGINE/ tools**	**Analytic methods/ algorithms**	**Visual Engine/ tool**	**Visual PRESENTATION**	***Specific features***	***Interactivity features***
Maciejewski et al, 2010 [107]	Indiana State cancer Registry; dataset details not provided.	Single dataset/database.	x				x						Algorithm is designed to identify hot and cold spots in mapped data by assessing the spatial association of a particular mapped unit to its surrounding units.	AMOEBA (A Multidirectional Optimum Ecotope-Based Algorithm) procedure for reducing small areas by enlarging the area base over which the summary statistic can be calculated.	Not provided.	Chloropleth map; clustering map offering comparison of incidence rates.	Group data spatially	Temporal controls
Maciejewski et al, 2011 [79]	Census, geographical boundaries of counties; pandemic data as example (H1N1 mentioned)	Simulation data									x		D3, Jquery	Not mentioned.	D3, Jquery	Geo-referenced data on a map	Able to simulate spatiotemporal outbreak	Scroll through map, able to adjust parameters, filtering options
Marek et al, 2015 [108]	National Institute of Public Health, Czech Republic data on 100,000 cases of Campylobacteriosis.	Single dataset/database.	x				x		x				R with spacetime, gstat and plotKML	Generating KML files using R with spacetime, gstat and plotKML.	Google Earth	1) Spatio-temporal bubble chart 2) Continuous spatio-temporal surface 3) Empirical spatio-temporal variogram and fitted theoretical spatio-temporal variogram	Time scale and location selection.	Scale and time interval
Mitrpanont et al, 2017 [109]	1,921 medical and public health research projects	Multiple dataset	x						x				Javascript, SQL	SQL for data querying.	D3 and C3 (Javascript)	Dashboard - including bar chart, radar chart, guage chart, word cloud, chloropleth map, multi-relationship network graph	Location specific details on each vis.	Slider bar to bottom of bar chart and radar allows user to select further options. Hovering over certain sections allows further visualizations

Author and year	DATA TYPE AND SOURCE												ANALYTIC METHODS		VISUALIZATION METHODS			
	**Data used and user case/s**	**Data sources**	**Structured**	**Unstructured**	**Semi-structured**	***Registry***	***Administrative/ survey***	***EMR/ EHR***	***ST***	***Sensor***	***Simulation***	***Web/Social media***	**Analytic ENGINE/ tools**	**Analytic methods/ algorithms**	**Visual Engine/ tool**	**Visual PRESENTATION**	***Specific features***	***Interactivity features***
Mittelstadt et al, 2014 [110]	The FDA Adverse Event Reporting System data source - first quarter of 2011	Database	x			x							Not clear. Computes O.R.s to surface adverse events.	Algorithm to separate relevant drug reactions from irrelevant ones. "Our algorithm queries the databasefor all drug (d) - reaction (r) co-occurrences and measurestheir frequenciesf(d, r). Then it calculates the relevanceR(d, r) =f(d, r)/n(d) with the frequencyf(d, r) divided by the number of records n(d) containing drug d."	visual analysis tools, no name is stated	Pixel interface + interactive scatter plot + Treemap overview.	Mouse over option for further details. Drill down function allows user to see detailed info	User selects drug of interest and relevant reactions. Automatic process analysis low frequency events and creates visual highlighting which allows user to analyze further via drill down/filtering
Ozkaynak et al, 2015 [111]	134,596 pediatric asthma encounters pooled from 3 datasets	Multiple Datasets	x					x					EventFlow	Discreet Time Markov Chains	Eventflow	EventFlow: Graphical presentation of events DTMCs were utilized to quantitatively represent workflow patterns.	Temporal data patterns, aggregated data summaries	Not mentioned.
Park et al, 2018 [112]	7,410 posts and 132,599 associated comments that were made by 41,967 unique members from three subreddits: r/Anxiety, r/Depression, and r/PTSD, from Oct, 2015 to Dec, 2015	Reddit posts and comments		x								x	Python Reddit API Wrapper (PRAW)	K-means clustering	D3, Gephi, ForceAtlas2	Bubble chart and network visualization		Not mentioned.
Perer et al, 2015 [113]	1 year EMR record; 1,386 patients, 11,058 hyperlipidemic diagnosis and 20,693 medication events	Single dataset/database.		x	x			x					Python based.	Frequent pattern mining algorithm, with a greedy approach for Two-Way sorting to reduce number of events.	D3.js vis toolkit	Bubble chart using frequency of events; Sankey chart uses patterns: Diagnosis to Medication; Lab to Diagnosis to Medication; etc.	Patterns mainly on bubble charts; and flows using Sanky like diagrams.	Sequence of nodes and edges for users to pick; drill down to next level of hierarchy.

Author and year	DATA TYPE AND SOURCE<br>Data used and user case/s	Data sources	Structured	Unstructured	Semi-structured	*Registry*	*Administrative/ survey*	*EMR/ EHR*	*ST*	*Sensor*	*Simulation*	*Web/Social media*	ANALYTIC METHODS<br>Analytic ENGINE/ tools	Analytic methods/ algorithms	VISUALIZATION METHODS<br>Visual Engine/ tool	Visual PRESENTATION	*Specific features*	*Interactivity features*
Proulx et al, 2006 [114]	World Health Organization's avian flu disease outbreak news and events timeline	Single dataset/database.		x									nSpace	Triaging massive data and sense making	GeoTime	Analytical dashboard and visual display of several maps	Structuring and restructuring of search queries allow for a vast array of information to be examined and analyzed	User search queries, filtering, everything that is found can be moved into the Sandbox; save it and build a bigger picture.
Shaban-Nejad et al, 2017 [84]	Initial data source: about 25% of CMA of Montreal Canada of about 1 million people.	Multiple datasets	x	x			x						PostgreSQL	Case-detection algorithms designed to identify cases of a particular disease or condition based on longitudinal individual-level data	OwlAPI	Causal diagram for conditions and risks; prevalence over time.	Multiple risk factors can be explored at the population level.	Stratifying population under different dimensions, able to standardize indicators directly.
Soulakis et al, 2015 [115]	548 patient records with 5513 providers and interactions; Northwestern Medicine Bluhm Cardiovascular Institute's Enterprise Data Warehouse (EDW) using ICD9 codes.	Single dataset/database.	x					x					ETL scripts using MS SQL Server Management Studio; parsed and edited in Perl and Python; Further analyss in Gephi, R, Neo4J	Force Atlas, Fruchterman Reingold; heuristic community detection by Blondel et al; Clique member and sizxe using the kCliques algorithm in RBGL R package.	Gephi; Neo4J	Relationship graph (similar to social network graph) and bar plots	Ability to encode implicit and explicit interactions between patients and providers	Not mentioned.
Tate et al, 2014 [87]	CPRD (the clinical practice research datalink) database, 14 million patients with 5.4 million patients being alive and registered from 660 primary care practices; Diabetes population in the database.	Administrative linked database	x					x					SQL; based on 3 database model, the user database holding searches; cached results database; SQL database containing anonymised data.	Rapid search algorithms; specific not mentioned; being patented.	TrialViz; specific visualization engine and methods not mentioned.	Stacks and cards	Searches presented in different toggle screens; geogrpahical distribution; and box charts.	Users select queries according to the inclusion and exclusion criteria.
Tilahun et al, 2014 [88]	WHO global health observatory data repository, 1990-2010. Missing data were complimented by country specific official sources.	Single dataset/database.	x										Resource Development Framework for data representation, Fuseki triple store for storage and Svigler for vis.	Data coversion using Excel2RDF converter; enrichment using Dbpedia, Bio2RDF and LinkedCT.	Sgvizler	LOD-based health information representation, querying, and visualization	SPARQL query interface, able to interact with data using retrieval or visualization tool	User input

**Author and year**	**DATA TYPE AND SOURCE**												**ANALYTIC METHODS**		**VISUALIZATION METHODS**			
	**Data used and user case/s**	**Data sources**	**Structured**	**Unstructured**	**Semi-structured**	***Registry***	***Administrative/ survey***	***EMR/ EHR***	***ST***	***Sensor***	***Simulation***	***Web/Social media***	**Analytic ENGINE/ tools**	**Analytic methods/ algorithms**	**Visual Engine/ tool**	**Visual PRESENTATION**	***Specific features***	***Interactivity features***
Toddenroth et al, 2014 [116]	EMR of 79,704 inpatients from the local university hospital in 2011.	Single dataset/database.	x					x					R package for statistical simulation and graphics; RColorLibrary for color sequences.	Hierarchical clustering, rule mining	R package	Heatmap used to graph statistical associations. Further pairing attribute sets enhances graphical presentation.	Pallette details heat coloring; Dendrogram illustrated clustering and associations.	Not mentioned.
Torres et al, 2012 [117]	National Health and Nutrition Examination Survey (NHANES)	Single dataset/database.	x				x						Tool/base not mentioned. Machine learning.	K-means clustering. Cluster density, variance, and standard deviation for each cluster are computed using Welford's method. Recurrence and Pearsons correlation coefficient calculated.	Not mentioned.	Scatter plot with real-time bar graphs	Interactive real-time scatter plot matrix that outputs extra data to real-time changing horizontal bar graphs	Filters, pan and zoom.
Widanagamaachchi et al, 2017 [72]	Multiparameter Intelligent Monitoring in Intensive Care database of tens of thousands of patients, close to 27 GB. EHR data.	Single dataset/database.	x					x					Machine learning and statistical methods.	Hierarchical clustering and correlations; Personalized mortality prediction driven by EHR and a patient similarity metric.	Not mentioned.	Dashboard, timeline	Three views, patient, patient grouping, and patient progression	Change focus time step and time window. Attribute exploration, filtering, hilighting, hiding.
Xing et al, 2010 [91]	NHANES (National Health and Nutrition Examination Survey) data	Single dataset/database.	x				x						C# using Microsoft Visual Studio 2005	Interactive mining based on Murtagh, 1983. A survey of recent advances in hierarchical clustering algorithms. The Computer Journal, 26(4), 354-359.; Computing the statistical measurements of a disease dominating patterns;	C#	Hierarchical clustering dendrogram; disease dominating pattern.	Clickable sub-clusters displayed as bar graph series.	Clicking on clusters leads to more clickable details.

Author and year	DATA TYPE AND SOURCE												ANALYTIC METHODS		VISUALIZATION METHODS			
	**Data used and user case/s**	**Data sources**	**Structured**	**Unstructured**	**Semi-structured**	***Registry***	***Administrative/ survey***	***EMR/ EHR***	***ST***	***Sensor***	***Simulation***	***Web/Social media***	**Analytic ENGINE/ tools**	**Analytic methods/ algorithms**	**Visual Engine/ tool**	**Visual PRESENTATION**	***Specific features***	***Interactivity features***
Xu et al, 2013 [73]	National Center for Health Statistics, Health Indicators Warehouse - IOM dataset	Single dataset/database.	x										Correlation mining for strength of relationships and predictions.	Correlation matrix uses Peartson product-moment correlation coefficient; Level-of-detail algorithm that includes more detail as the analyst zooms in	D3	Heatmap visualization of a correlation matrix, color scale maps,	High level overview on pairwise correlations in heatmap form - zoomed in view of same correlations with increased detail - and a tagging view that allows documentation of comments	User tooltips, users can share findings and results by commenting directly with a tagging feature, drill down features
Yan et al, 2013 [118]	Pilot data used from Shayang and Qianjian with 9958 and 19641 reported symptoms.	Pooled data from various sources within the health system.	x				x		x				PHP 5.3.2	Shewhart Chart (P Chart), Moving Average (MA), Exponentially Weighted Moving Average (EWMA), Cumulative Sums (CUSUM)), spatial analysis (Recursive Least Square (RLS) Method, Small Area Regression and Testing (SMART), Bayesian spatial scan statistics), and spatial-temporal analysis (Space-time Scan Statistics and What is	Google Maps with dynamic layers from other symptoms	Line, bar, pie charts. Can choose to view data sources, geographic region, time intervals.	Real time visualization	Alerts information for one or more groups, presented chronologically, Provides plotting capabilities; user can select sources.
Yu et al, 2017 [82]	(1) Medicaid Analytic eXtract (MAX) file; (2) National Provider Identification (NPI); (3) Census and Medicaid enrollees' racial/ethnic composition; and (4) disease classification.	Multiple datasets	x						x				Data storage layer based on NoSQL, MongoDB; Server layer using HTML, CSS and Javascript; Client layer based on d3.js.	Algorithm used for information regarding geospatial population data, in the Replica view.	HTML, CSS, and Javascript	Geospatial using topological features, concurrent interactive chloropleth maps	Disease specific visualizations	Capability to view and interact with several screens concurrently at a larger scale., filtering functionality, clickable features

Author and year	DATA TYPE AND SOURCE												ANALYTIC METHODS		VISUALIZATION METHODS			
	**Data used and user case/s**	**Data sources**	**Structured**	**Unstructured**	**Semi-structured**	***Registry***	***Administrative/ survey***	***EMR/ EHR***	***ST***	***Sensor***	***Simulation***	***Web/Social media***	**Analytic ENGINE/ tools**	**Analytic methods/ algorithms**	**Visual Engine/ tool**	**Visual PRESENTATION**	***Specific features***	***Interactivity features***
Yu et al, 2018 [74]	Datasets pertaining to Beijing for three primary categories, air pollution (PM2.5 concentration), environmental factors (temperature and season change), and respiratory diseases (emergency room visits- total and disease specific; also worldwide exposure data for PM2.5.	Multiple datasets	x									x	IBM Watson Analytics	Not mentioned.	IBM Watson Analytics	Descriptive statistics and trends over time.	Chloropleth maps, bar graphs	Pan, zoom, other functions visible on graphics; not mentioned in the article.

x = applicable category
