# Supplementary material for: Visual Analytic Tools and Techniques in Population Health and Health Services Research: Scoping Review
Source: J Med Internet Res. 2020 Dec 3;22(12):e17892. doi: 10.2196/17892 (PMC7716797; doi:10.2196/17892)
Supplement: Multimedia Appendix 6 [file jmir_v22i12e17892_app6.pdf]

Multimedia Appendix 6: Domains of Healthcare	
**Population Health**	
Clinical Populations	Abusharekh et al, 2015 [67]; Antoniou et al, 2010 [93]; Baytas et al, 2016 [80]; Benis et al, 2017 [89]; Castronovo et al, 2009 [77]; Chen et al, 2016 [95]; Chorianopoulos et al, 2016 [96]; Dagliati et al, 2018 [66]; Gligorijevi et al, 2017 [98]; Gotz et al, 2014 [76]; Haque et al, 2014 [99]; Huang et al, 2015 [101]; Hund et al, 2016 [90]; Jiang et al, 2016 [103]; Kaieski et al, 2016 [104]; Lu et al, 2017 [71]; Marek et al, 2015 [108]; Mitrpanont et al, 2017 [109]; Mittelstadt et al, 2014 [110]; Ozkaynak et al, 2015 [111]; Park et al, 2018 [112]; Perer et al, 2015 [113]; Tate et al, 2014 [87]; and Toddenroth et al, 2014 [116].
Demographic Populations	Haque et al, 2014 [99]; Hardisty et al, 2010 [100]; Jiang et al, 2016 [103]; Jinpon et al, 2017 [83]; Katsis et al, 2017 [105]; Kruzikas et al, 2014 [106]; Lu et al, 2017 [71]; Maciejewski et al, 2010 [107]; Marek et al, 2015 [108]; Mitrpanont et al, 2017 [109]; Mittelstadt et al, 2014 [110]; Perer et al, 2015 [113]; Shaban-Nejad et al, 2017 [84]; Tate et al, 2014 [87]; Xing et al, 2010 [91]; and Xu et al, 2013 [73].
Epidemic Monitoring and Modelling	Ali et al, 2016 [68]; Alonso et al, 2012 [92]; Bryan et al, 2015 [64]; Byrd et al, 2016 [94]; Castronovo et al, 2009 [77]; Chen et al, 2016 [95]; Chorianopoulos et al, 2016 [96]; Deodhar et al, 2015 [65]; Guo et al, 2007 [69]; Hardisty et al, 2010 [100]; Ji et al, 2012 [102]; Ji et al, 2013 [81]; Kostkova et al, 2014 [75]; Luo et al, 2016 [78]; Maciejewski et al, 2011 [79]; Proulx et al, 2006 [114]; Tilahun et al, 2014 [88]; and Yan et al, 2013 [118].
Spatiotemporal	Ali et al, 2016 [68]; Alonso et al, 2012 [92]; Bryan et al, 2015 [64]; Byrd et al, 2016 [94]; Castronovo et al, 2009 [77]; Chen et al, 2016 [95]; Chorianopoulos et al, 2016 [96]; Deodhar et al, 2015 [65]; Guo et al, 2007 [69]; Hardisty et al, 2010 [100]; Ji et al, 2012 [102]; Ji et al, 2013 [81]; Kostkova et al, 2014 [75]; Luo et al, 2016 [78]; Maciejewski et al, 2011 [79]; Proulx et al, 2006 [114]; Tilahun et al, 2014 [88]; and Yan et al, 2013 [118].
GIS	Kaieski et al, 2016 [104] and Tilahun et al, 2014 [88].
**Health Services**	
Access	Gligorijevi et al, 2017 [98]; Haque et al, 2014 [99]; Jiang et al, 2016 [103]; Jinpon et al, 2017 [83]; Lavrac et al, 2007 [70]; Marek et al, 2015 [108]; Mitrpanont et al, 2017 [109]; Soulakis et al, 2015 [115]; Tate et al, 2014 [87]; and Yu et al, 2017 [82].
Utilization	Abusharekh et al, 2015 [67]; Jiang et al, 2016 [103]; Jinpon et al, 2017 [83]; Katsis et al, 2017 [105]; Kruzikas et al, 2014 [106]; Marek et al, 2015 [108]; Mitrpanont et al, 2017 [109]; Ozkaynak et al, 2015 [111]; Perer et al, 2015 [113]; Soulakis et al, 2015 [115]; Tate et al, 2014 [87]; Toddenroth et al, 2014 [116]; Widanagamaachchi et al, 2017 [72]; and Yan et al, 2013 [118].
Costs	Kruzikas et al, 2014 [106]; and Mitrpanont et al, 2017 [109].
