# Supplementary material for: Visual Analytic Tools and Techniques in Population Health and Health Services Research: Scoping Review
Source: J Med Internet Res. 2020 Dec 3;22(12):e17892. doi: 10.2196/17892 (PMC7716797; doi:10.2196/17892)
Supplement: Multimedia Appendix 7 [file jmir_v22i12e17892_app7.pdf]

| Author and year | Key findings | Innovation and impact of the VA method/uptake of the method | Limitations | In use at the time of publication | Application publicly available | Tools free or open source | Application developed in consultation with the target audience |
|---|---|---|---|---|---|---|---|
| Abusharekh et al, 2015 [67] | H-DRIVE is a powerful data analytic and viz tool for various scenarios, offering a wide variety of analytic capabilities. User case shows the use of descriptive and predictive modeling ability towards a common utilization scenario. | Intuitive and powerful analytics platform to derive contextualized, customized, comprehensive, consumable and clear strategic intelligence and situational awareness. | Not mentioned | Yes. | Not applicable. | Not applicable. | Not mentioned. |
| Afzal et al, 2011 [85] | Support tool for epidemic modeling, and studying various scenarios using different models and interventions. | Decision history visualization and navigational support helps the users analyze the consequences of their decisions over time and understand both the short term and long term impact of their mitigative responses; Tool also offers drill down ability. | Not mentioned. | Not applicable. | Not applicable. | Not mentioned. | Not mentioned. |
| Ali et al, 2016 [68] | Tool from a lower middle income country using multiple data sources to vis and real time monitoring; with predictive analytic ability. | Tool with various modules to process data from various sources for ID modeling and vis. | Not mentioned | Yes. | No | No | Not mentioned. |
| Alonso et al, 2012 [92] | EPIPOI is able to handle complex analyses. A prototype has been used to assist researchers in a variety of contexts. | Enables epidimeologic time series analysis with enhanced capability of reproducible (flexibility of using on multiple datasets). | Does not cover possibilities such as reproductive number of a pathogen; does not allow the inclusion of additional variables. | Yes. | Not mentioned. | Yes | Yes |
| Antoniou et al, 2010 [93] | Tool for querying global studies focused on 4 major autoimmune conditions with population health metrics such as mortality. | Brings together publications and data on 4 major autoimmune conditions. | Not mentioned. | Yes. | Yes | Not mentioned. | Not mentioned. |
| Antunes de Mendonca et al, 2015 [86] | Case study demonstrates that government data visualization is possible and an easy way for the public to receive information. | Using Brazil's open government data to inform citizens about epidemic tracking | Government resistance to the use of open data; data harmonization an issue. | Not applicable. | Not applicable. | Yes. | Not mentioned. |
| Baytas et al, 2016 [80] | Results show that PHENOTREE can detect clinically meaningful hierarchical phenotypes. | Includes aspects of unsupervised mining of data; adds multiple layers rather than linear relations. | Dependence on clinicians and users to specify features in datasets. Otherwise, PhenoTrees may become noisy or irrelevant. | Not applicable. | Not applicable. | Not applicable. | Not mentioned. |
| Benis et al, 2017 [89] | Tool to select database of choice and generating heatmaps for correlations. | Allows users to deal with very large datasets by reducing their complexity, being able to be easily displayed in a visual format, using hierarchical clustering. | Not mentioned. | Not mentioned. | Not mentioned. | Yes. | Not mentioned. |

| Author and year | Key findings | Innovation and impact of the VA method/uptake of the method | Limitations | In use at the time of publication | Application publicly available | Tools free or open source | Application developed in consultation with the target audience |
|---|---|---|---|---|---|---|---|
| Bryan et al, 2015 [64] | A powerful tool for epidemic simulation and predictive analytics. | Combination of a number of statistical metrics, interactions, predictive models, a novel spatial predictor, and visualization techniques for emulator building, usage, and analysis in one tool. | While building the predictive model, real time effects, correlations and results cannot be viewed. | Yes. | Not applicable. | Not applicable. | Yes |
| Byrd et al, 2016 [94] | Aggregated tweets on influenza using open source tool that provides replicability. | Method to extract, classify and map twitter data, based on sentiment analysis, using NLP. | Limited to selected cities, and lower predictive levels. | Yes. | Yes. | Yes. | Not mentioned. |
| Castronovo et al, 2009 [77] | Dynamic mapping provides a practical visualization tool to examine seasonality in infectious disease and its relationship to environmental factors. | Proposed new method for seeing changes in disease dynamics using dynamic maps. | Does not emphasize specific rates for specific locations and time periods | Not applicable. | Not applicable. | Not applicable. | Not mentioned. |
| Chen et al, 2016 [95] | Designed an online scan statistics tool to prospectively detect active dengue fever hot spots and tested it. | Online tool to predict dengue fever hot spots. | Did not consider the true commuting flow patterns due to data unavailability at the village level. | Not applicable. | Not applicable. | Not mentioned. | Not mentioned. |
| Chorianopoulos et al, 2016 [96] | Flu symptom tweets are displayed on a map available to the public. First application with an open source code, also allowing for linked data between various applications. | Only flu-symptom mapping application to be open-sourced and fully available to the public. | Ineroperability a main concern for the field; transparency and interoperability would offer better benefits to public health. | Yes. | Yes | Yes. Source code. | Not mentioned. |

| Author and year | Key findings | Innovation and impact of the VA method/uptake of the method | Limitations | In use at the time of publication | Application publicly available | Tools free or open source | Application developed in consultation with the target audience |
|---|---|---|---|---|---|---|---|
| Dagliati et al, 2018 [66] | Unique dashboard for diabetes clinical decision making and population level predictive modeling for complications. | Integrates results from research on previously collected data to support clinical decision-making, predictive models and advanced temporal data-mining solutions previously validated on routinely collected clinical and administrative data. | Not possible to assess clinical outcomes; some core features rely on an agreement among several health care institutions to share data; tool evaluated in a diabetes specialty clinic, limiting generalizability | Not applicable. | Not applicable. | Not applicable. | Not mentioned. |
| Deodhar et al, 2015 [65] | Presented EpiCaster, an integrated system for situation assessment and forecasting of several global epidemics. | Reduces the timeline for presenting results of forecasts on the Web for any new disease, or an existing disease like Flu in a new region. | Not mentioned. | Yes. | Not applicable. | Not applicable. | Not mentioned. |
| Garcia-Marti et al, 2017 [97] | Human-related factors are more important to model tick bites than using environmental variables that characterize the environmental conditions of a given location for a particular time frame. | First study that applies the frequent pattern mining method to a volunteered dataset that pertains to the public health domain. | Improved forecasting model for tick bite risk needed. | Not applicable. | Not applicable. | Not applicable. | Not mentioned. |
| Gligorijevi et al, 2017 [98] | The presented system is a prototype with capability to be used for planning the optimization and improvement of an entire healthcare system. | Prototype developed to visualise large EHR datasets, | Machine learning techniques to be integrated in future. | Not applicable. | Not applicable. | Not applicable. | Not mentioned. |
| Gotz et al, 2014 [76] | New visual exploratory method for visually querying EMR databases for patterns related to preconditions, events and outcomes. Application is in development, and do not provide enough details for replicability. | Provides an interactive visual environment for the exploration and analysis of temporal medical event data. | Method assumes linearity of events; does not distinguish between similar patients. | Not applicable. | Not applicable. | Not applicable. | Not mentioned. |
| Guo et al, 2007 [69] | The discovered spatial interaction patterns provide valuable insight for designing effective pandemic mitigation strategies and supporting decision-making in time-critical situations. | The approach combines graph partitioning, linear ordering, matrix-based visualization, and flow maps to synthesize, visualize, and interpret spatial interaction patterns. | Dynamic, real time data is left for future. Interaction with demographic data not taken into account. | Not applicable. | Not applicable. | Not applicable. | Not mentioned. |
| Haque et al, 2014 [99] | Demonstrated BI tools used to monitor healthcare for decision making. | Data analytic tool for monitoring access and utilization for a rural health service in remote settings. | Not intended for real-time data; | Yes. | Not applicable. | Not applicable. | Not mentioned. |
| Hardisty et al, 2010 [100] | Innovative tool for studying spatio temporal associations to monitor epidemics. | Demonstrates the use of space-time correlations to monitor epidemics. | Not mentioned. | Not mentioned. | Not applicable. | Not applicable. | Not mentioned. |

| Author and year | Key findings | Innovation and impact of the VA method/uptake of the method | Limitations | In use at the time of publication | Application publicly available | Tools free or open source | Application developed in consultation with the target audience |
|---|---|---|---|---|---|---|---|
| Huang et al, 2015 [101] | A visual mining system to support exploratory data analysis of multi-dimensional categorical EMR data, using CKD as a model disease. | Visual mining system for exploratory data analysis, without the user having programming knowledge. | Overplotting, selection of risk factors given the high number in the dataset. | Not applicable. | Not applicable. | Not applicable. | Not mentioned. |
| Hund et al, 2016 [90] | SubVIS overcomes problems of subclustering algorithms, the user case is of a small dataset of older adults with influenza. | Overcomes challenges of subspace clustering algorithms, namely (1) difficult reault interpretation (2) redundancy of detected subspaces and clusters, and (3) different clustering results for different parameter settings. | Does not account for non-linear relationships; for clustering dimensions. | Not applicable. | Not applicable. | Not applicable. | Not mentioned. |
| Ji et al, 2012 [102] | Tool enables users to monitor public health concern over time and through a set of visualization tools for epidemics-related Twitter data. | Innovative tool for analysing twitter data for epidemic monitoring and prediction; modules clean noisy geographic locations and identify levels of granularity for different location specifications in 3 different visualizations. | Not mentioned. | Not mentioned. | Not mentioned. | Not mentioned. | Not mentioned. |
| Ji et al, 2013 [81] | Epidemic Sentiment Monitoring System (ESMOS) provides tools for visualizing Twitter users' concern towards different diseases for public health officials for preventive actions. | One of the few tools offering analytics for social media epidemic monitoring. | Not mentioned. | Not mentioned. | Not mentioned. | Not mentioned. | Not mentioned. |
| Jiang et al, 2016 [103] | Tool developed for integrating geo-spatial and multi attribute patient data, over time. | Creating two new visualization methods for displaying healthcare data; integrating patient attributes and ge-spatial data. | Contouring must fit to certain region or else the visual becomes unclean and inaccurate; refinement of the tool is needed. | Yes. | Not mentioned. | Not mentioned. | Yes |
| Jinpon et al, 2017 [83] | Integrated visualization tool promoting understanding of community well-being status and enhancing health decision-making in Thailand. Tool in use by health managers. | Tool being used for decision-making because of the ease of access, computation via a user-friendly interface, and visualizion showing performance metrics and other aggregate data. | Not mentioned. | Yes. | No | Yes. | Not mentioned. |
| Kaieski et al, 2016 [104] | Vis-Health uses an established statistical technique for visualizing correlations between different variables, and is available as an open tool. | Tool shows correlations of variables, user case on dengue and climatic factors, overlaid on a map. Open source tools mentioned. | Not mentioned. | Not mentioned. | Yes. | Yes. | Not mentioned. |
| Katsis et al, 2017 [105] | The resulting system will be an important tool for public health researchers, that will help them make interesting discoveries from the continuously increasing amounts of health-related data. | Study leveraged ML techniques for exploring public health and related datasets; tested multiple combination of techniques. | Tool in development. | Not mentioned. | Not mentioned. | Not mentioned. | Not mentioned. |

| Author and year | Key findings | Innovation and impact of the VA method/uptake of the method | Limitations | In use at the time of publication | Application publicly available | Tools free or open source | Application developed in consultation with the target audience |
|---|---|---|---|---|---|---|---|
| Kostkova et al, 2014 [75] | Simulation with three datasets from the swine flu 2009 pandemic (HPA surveillance, Google news, Twitter) demonstrates the potential of medi+board to automate data processing and visualization to assist public health experts in decision making on control and response measures. | A potential public health tool for screening real time data. | Not mentioned. | Not mentioned. | Not mentioned. | Not mentioned. | Not mentioned. |
| Kruzikas et al, 2014 [106] | Integration of an agent-based modeling with data visualization can inform complex health care infrastructure investment decisions. | Study considers economic and longer term impact of various decisions on a major condition of interest. | Not mentioned. | Not mentioned. | Not mentioned. | Not mentioned. | Not mentioned. |
| Lavrac et al, 2007 [70] | Developed methods and tools to help regional public health institutes and the national Institute of Public Health to perform more effectively. | Innovative use of data mining and visualization for decision support in planning and management of Slovenian public health-care. | Not mentioned. | Not mentioned. | Not mentioned. | Not mentioned. | Not mentioned. |
| Lu et al, 2017 [71] | Proved to be powerful in identifying episode sequencing patterns which were consistent with real-world clinical outcomes. | Developed framework for health data anaytics with capability to integrate mutiple database. Retrospective analysis of cancer EPR/EHR (from 1970s) to suggest improvements in treatment. | Many aspects that not automated. Database pre-processing, warehousing, analytics, mining, timelines utilize different, human dependent | Not mentioned. | Not mentioned. | Yes. Weka is open source and free | Not mentioned. |
| Luo et al, 2016 [78] | Number of rings and the number of enumeration units (e.g., counties) can be represented in a single ring map are limited, both from a practical design standpoint and in terms of user comprehension. | Proposes a new framework in terms of effective disease control that starts from identifying geosocial interaction patterns, followed by designing effective control measures accordingly, and then evaluates the efficacy of different control measures. | Spatial proximity measures do not include topology; vaccination strategies difficult to extrapolate to complex datasets. | Not mentioned. | Not mentioned. | Yes. Source code | Not mentioned. |
| Maciejewski et al, 2010 [107] | Exploratory tool for hypotheses generation and knowledge discovery, displaying trends and patterns in data, without applying statistics for significance. | Offers VA tool for targeting the problem of small areas and numbers that may skew results; and that have been traditionally dealt with using suppression. | Not mentioned. | Not mentioned. | Not mentioned. | Not mentioned. | Not mentioned. |
| Maciejewski et al, 2011 [79] | Educational tool in development, and available for epidemic modeling and visualization. | Educational tool for simulating epidemic scenarios. | Emergency response tool in development, and would incorporate better analytic techniques. | Yes. | Yes. | Yes. | Yes. |

| **Author and year** | **Key findings** | **Innovation and impact of the VA method/uptake of the method** | **Limitations** | **In use at the time of publication** | **Application publicly available** | **Tools free or open source** | **Application developed in consultation with the target audience** |
|---|---|---|---|---|---|---|---|
| Marek et al, 2015 [108] | Using geovisual analytics authors were able to display and retrieve information from complex dataset efficiently. | Combines geo-spatial disease data with Google Earth, a freely accessible tool. | Google Earth has to be coupled with an analytic tool. | No. | No. | Yes. | Not mentioned. |
| Mitrpanont et al, 2017 [109] | SAGE2 is a tool that allows MedThaiVis to be expanded upon to larger high-resolution displays allowing for more information display and remote collaboration | Tool that allows greater intake of information using MedThaiVis tool. | Useability testing for effectiveness needed; planned. | Yes. | No. | No. | Not mentioned. |
| Mittelstadt et al, 2014 [110] | A visual analytics tool that receives user input regarding selection of drugs and through signal processing showcases low frequency reactions that may have been overshadowed otherwise. | Tool to examine low frequency drug reactions often missed by other analyses. Offers scalable interfaces and interactive options. | Not mentioned. | Yes. | Not applicable. | Not applicable. | Yes |
| Ozkaynak et al, 2015 [111] | EHR data (and utilization of visual analytics and Markov Chains) and field data can be used together in a sequential design for a richer understsanding of clinical workflow. | The workflow analysis incorporates probabalistic and trends to propose improvements that are replicable and presents data visually. These are not possible using traditional qualitative techniques for clinical flow | Findings need to be complemented with field observations. | Not applicable. | Not applicable. | Not applicable. | Not mentioned. |
| Park et al, 2018 [112] | Compared online discussion content from three online mental health communities concerning conditions that similar symptoms and can potentially be co-morbid. | Use of Reddit data for discussion on mental health topics. | Visualizations and findings are limited to the discussions; online discussions change over time. | Not applicable. | Not applicable. | Not applicable. | Not mentioned. |
| Perer et al, 2015 [113] | Tool for exploring patient care pathways from EMR data, mine patterns and constructing flow diagrams. | Patient care pathways can be explored in EMR data. | Scalability with the algorithm; large datasets will slow down the system. Support thresholds need more work. | Not mentioned. | Not mentioned. | Not mentioned. | Not mentioned. |
| Proulx et al, 2006 [114] | nSpace and GeoTime are tools allowing hypothesis creation and insights into unstructured data. | Allows for unstructured information to be visualized into geo-spatial insights. | Seamless transfer of data from one environment to another, and from unstructured to structured environments. | Yes. | Not applicable. | Not applicable. | Yes |
| Shaban-Nejad et al, 2017 [84] | Described the PopHR platform and discuss the architecture, design, key modules, and its implementation and use. | Population level, multi-data set tool for exploration of conditions and risk factors, based on social determinants of health approach. | Prototype, which will be scaled up. | Not applicable. | Not applicable. | Not applicable. | Not mentioned. |

| Author and year | Key findings | Innovation and impact of the VA method/uptake of the method | Limitations | In use at the time of publication | Application publicly available | Tools free or open source | Application developed in consultation with the target audience |
|---|---|---|---|---|---|---|---|
| Soulakis et al, 2015 [115] | Case study of patients with heart failure indicates that healthcare providers have a strong tendency to access healthcare records for common sets of patients, indicating likely collaboration with other providers. | Network analysis provides strong evidence of interactions across teams of providers. | Results do not take into account length of stay; would require further refinement through normalization. | Yes. | Not mentioned. | Not mentioned. | Not mentioned. |
| Tate et al, 2014 [87] | The TrialViz system identifies individuals potentially available for study recruitment, representing a significant advantage over other systems. | Most SQL-based systems that exist at present are not capable in general of doing this. | Not mentioned. | Yes. | Not applicable. | No. | Yes. |
| Tilahun et al, 2014 [88] | LOD has a promising potential in the representation of complex health-related data. This is mainly due to its reusable and interoperable manner that can serve intelligent queries, and ultimately support decision making. | Evaluates the use of linked open data for global prevalence of HIV, which can be used as a model for other conditions and user cases. | Semantic Web technologies work well with small datasets but might not be the best option with big | Not applicable. | Not applicable. | Not applicable. | Yes. |
| Toddenroth et al, 2014 [116] | Proposed approach to use of heat maps for studying associations and causality; based on clustering. | Using heat maps for predictive purposes - selectively pairing different attributes allows for more meaningful presentation of results. Clustering algorithm can partly rearrange attributes to accomplish this as well. | Causality cannot be established using the tool. | Not applicable. | Not applicable. | Not applicable. | Not mentioned. |
| Torres et al, 2012 [117] | Using an interactive scatterplot, the user is able to explore a complex and multidimensional dataset, with visuals that automatically rearrange to present most useful information first. This aids in hypothesis formation and decision support. | Allowing a real-time dynamic examination of survey result interactions | Improvements suggested; adding an interative exploratory component. | Yes. | Not mentioned. | Not mentioned. | Not mentioned. |
| Widanagamaachchi et al, 2017 [72] | Use of patient progression monitoring using ICU data, with health service metrics such as length of stay; predictive ability as well. | Researchers are able to explore patient groups and progress over time and to identify frequently used progression paths - while being able to refer back to the workspace of data for a visual display | Similarity metrics need to be improved. | Not mentioned. | Not mentioned. | Not mentioned. | Yes. |
| Xing et al, 2010 [91] | The results on the latest NHANES data demonstrate that these methods can mine meaningful disease associations consistent with the existing knowledge and literatures | Applied disease pattern mining tools for interactive clustering and graphs. | Basic visualzation. Authors mention more statistical analysis and visualisations for future. | Yes. | Yes. | Yes. | Not mentioned. |
| Xu et al, 2013 [73] | Using a national health database for indicators of health, this tool aggregates and correlates various indicators and their results into a heatmap-style display. This display can also be used by several analysts at the same time and publically commented upon for collaboration. | Using large databases to test associations within different variables and sub-variables. | Correlation statistical method assumes linearity. | Yes. | Yes. | Yes. | Not mentioned. |

| Author and year | Key findings | Innovation and impact of the VA method/uptake of the method | Limitations | In use at the time of publication | Application publicly available | Tools free or open source | Application developed in consultation with the target audience |
|---|---|---|---|---|---|---|---|
| Yan et al, 2013 [118] | Pilot study for the implementation of a survellance system for outbreak detection; validation required with real data. | Surveillance system, complemented with existing systems for data collection. | Further validation required to formally assess capability for early detection of incidents. | Yes. | No. | No. | Not mentioned. |
| Yu et al, 2017 [82] | Proposed a tool for healthcare policy maker and hospital admin to examine health care providers and the accessibility of care. | Tool for targeting access concerns using multiple datasets, and generating visualizations. | Does not include certain hospital info such as cost, payments etc. | Not mentioned. | Not mentioned. | Not mentioned. | Yes. |
| Yu et al, 2018 [74] | Findings using this tool can provide direct supports for strategy/decision making in public health and emergency administration. | Analytic tool capable of querying, correlating findings and predictive modeling from multiple data sources, including social media. | Not mentioned. | Not mentioned. | Not mentioned. | No. | Not mentioned. |
