# Supplementary material for: Visual Analytic Tools and Techniques in Population Health and Health Services Research: Scoping Review
Source: J Med Internet Res. 2020 Dec 3;22(12):e17892. doi: 10.2196/17892 (PMC7716797; doi:10.2196/17892)
Supplement: Multimedia Appendix 8 [file jmir_v22i12e17892_app8.docx]

# Multimedia Annex 8: Checklist for reporting visual analytic applications in population health and health services research (fillable)**.**

| Item | | Explanation | Section reported and comments |
| --- | --- | --- | --- |
| **A. User case, objectives, and goals of the application** | | | |
|  | **1. Setting** | | |
|  |  | a. Provided the setting for the study (e.g. government ministry, health unit, academia, industry, or a collaboration of settings) |  |
|  |  | b. Described where the study idea was initiated and if it was due to a specific need-based research question |  |
|  | **2. Target audience** | | |
|  |  | a. Described the intended target audience of the application (e.g. population or public health practitioners, clinicians, data scientists, industry, public and patient groups, policy and decision makers) |  |
|  |  | b. Discussed whether there were any changes in the target audience once the application was developed |  |
|  | **3. User case(s)** | | |
|  |  | a. Indicated the problem addressed |  |
|  |  | b. Described the objectives and whether they related to the settings and target audience |  |
|  |  | c. Provided the context (e.g. scoping exercise, prototype development, proof of concept, evaluation of a product, or a combination) |  |
|  | 4. Participatory design or development, co-design | a. Indicated whether the tool was co-designed or evaluated with key stakeholders |  |
|  | **5. Analytic capability** | | |
|  |  | a. Indicated whether the application was designed for descriptive, predictive or prescriptive analytics, visual exploration of complex datasets, or a combination |  |
|  |  | b. Described how the application was used to develop the application further (e.g. a descriptive analytics application extended towards a predictive one) |  |
|  | 6. Goal of the application or method | a. Discussed whether the application was geared towards exploratory analysis for knowledge discovery, decision support, or both |  |
| **B. Analytic and visualization methods** | | | |
|  | **7. Data source and type** | | |
|  |  | a. Provided the number of data sources (single or multiple) |  |
|  |  | b. Indicated the type of health care data (e.g. registry, administrative or national survey, electronic health or medical record, spatiotemporal or geospatial or gis, sensor, simulation, web sites, social media) |  |
|  |  | c. Indicated whether the data were structured, unstructured, or semi-structured |  |
|  |  | d. If applicable, for ‘smart’ devices (e.g. smartphone, health watch), indicated whether it was from an app, built-in device (e.g. GPS, gyroscope), an extended device (e.g. heart monitor), or a combination |  |
|  | **8. Hardware requirements and software tools** | | |
|  |  | a. Indicated the hardware for developing and running the application |  |
|  |  | b. Discussed the software environment needed to run the application |  |
|  |  | c. Provided information on the tools used to develop the visual and analytic engines (established engines, proprietary tools, versions for development, or other important details) |  |
|  | **9. Analytic, statistical and machine learning or artificial intelligence methods** | | |
|  |  | Described the analytic methods in detail including aspects of:    a. Data pipeline and figure of the pipeline (e.g. extract, transform and load (ETL) engine) |  |
|  |  | b. Data manipulation, cleaning, accuracy, quality issues |  |
|  |  | c. Querying, transformation |  |
|  |  | d. Limitations of the data collection and querying system |  |
|  |  | e. Methods of analysis, along with specific details on statistical and machine learning methods |  |
|  |  | f. Reasons for selecting the appropriate method applied to the user case |  |
|  |  | g. Contrasting other available methods |  |
|  |  | h. Machine learning algorithms |  |
|  |  | i. Source code repository |  |
|  |  | k. Analytic engine (part of the visualization tool used; a combination of tools or packages) |  |
|  | **10. Visual presentation(s)** | | |
|  |  | a. Indicated which metrics were being visualized and the aggregation methods used |  |
|  |  | b. Provided the platforms for presentation (e.g. website, dedicated app) |  |
|  |  | c. Detailed the devices (e.g. cross platform, cross device such as tablets and computers) |  |
|  | **11. Interactivity features** | | |
|  |  | a. Discussed the interactivity features (e.g. connections between multiple items simultaneously presented, drill downs, sliced and diced visualizations) |  |
|  |  | b. Discussed the types of interactivity (e.g. mouse hover, breakdowns, clickability) |  |
|  | 12. Specific features | Indicated the distinguishing features, especially in comparison to previous versions and similar applications |  |
| **C. Healthcare domain, availability and impact** | | | |
|  | **13. Domains of healthcare** | | |
|  |  | Provided details on the domains of healthcare addressed:  a. Population health: population demographics, one or more clinical conditions  b. Areas of epidemiology: spatiotemporal, geographic information system (GIS)  c. Areas of health services research: access, utilization, costs |  |
|  | **14. Uptake and availability of the tools and applications** | | |
|  |  | a. Indicated whether the application was in use |  |
|  |  | b. Indicated whether the tools used to build the application were open source, available on the web, or proprietary |  |
|  | **15. Innovation and impact of the visual analytic method or uptake of the method** | | |
|  |  | a. Discussed the improvements in the method(s), tool(s) and application(s) used over those previously used |  |
|  |  | b. Provided the lessons learned in the process of development |  |
|  |  | c. Discussed the impact on the settings, target audience, and methods |  |
|  | **16. Strengths, limitations and suggestions for further development** | | |
|  |  | a. Provided the strengths and limitations related to different aspects of the method(s), tool(s) and application(s) used |  |
|  |  | b. Offered recommendations regarding the adaptation and further development of the tool(s) |  |
